# Prescription Drug Monitoring Program Reminder Emails, Program Use, and Prescribing: A Randomized Clinical Trial

**DOI:** 10.1001/jamahealthforum.2025.5623

**Published:** 2025-12-19

**Authors:** Adam Sacarny, Tatyana Avilova, Ian Williamson, Weston Merrick, Mireille Jacobson

**Affiliations:** 1 Department of Health Policy and Management, Mailman School of Public Health, Columbia University, New York, New York; 2 National Bureau of Economic Research, Cambridge, Massachusetts; 3 Abdul Latif Jameel Poverty Action Lab, Cambridge, Massachusetts; 4 Secretariat, Washington, DC; 5 Minnesota Management and Budget Agency, Saint Paul; 6 Schaeffer Center for Health Policy & Economics, University of Southern California, Los Angeles; 7 The Leonard Davis School of Gerontology, University of Southern California, Los Angeles

## Abstract

**Importance:**

Prescription Drug Monitoring Programs (PDMPs) can contribute to safer prescribing of opioids and other controlled substances but are often underutilized by prescribers. Evidence on the feasibility and effects of email campaigns to increase clinicians’ engagement with PDMP is limited.

**Objective:**

To investigate whether email communications can increase PDMP use and, in turn, reduce guideline-discordant prescribing among clinicians.

**Design, Setting, and Participants:**

This randomized clinical trial included clinicians who prescribed controlled substances but lacked PDMP accounts, had inactive PDMP accounts, did not search the PDMP, or searched infrequently when prescribing opioids. The study was conducted from January 2024 to November 2025 in the US state of Minnesota.

**Interventions:**

Clinicians were randomized (1:1:1 ratio) to 2 intervention groups or a usual care group. Clinicians in the intervention groups were sent up to 2 emails highlighting their lack of PDMP engagement (no account, inactive account, no search, or infrequent search) with hyperlinks to the PDMP website to rectify it. Email content differed by group to focus on either the state’s legal requirements to use the PDMP or the clinical benefits of PDMP use.

**Main Outcomes and Measures:**

PDMP engagement, defined as engaging in the activity encouraged in the email (account creation, account reactivation, database searches, or more frequent database searching); and guideline-discordant opioid prescribing, a composite of 5 prescribing measures, both measured within 60 days of the initial email. Secondary outcomes included email communication engagement and components of both primary end points.

**Results:**

Among 7872 clinicians, 6574 were physicians (83.5%) and 4385 were men (55.7%). PDMP engagement was 11.8% (309 clinicians) in the usual care group. Legal requirement emails raised the engagement rate by 26.5 (95% CI, 24.3-28.7; *P* < .001) percentage points, while clinical benefit emails raised engagement 14.2 (95% CI, 12.0-16.3; *P* < .001) percentage points. Both types of emails raised account holding, database searches, and searches for patients with a history of risky prescribing. Effects endured for at least 7 months. Guideline-discordant prescribing did not differ significantly across groups.

**Conclusions and Relevance:**

In this randomized clinical trial, email communications increased PDMP engagement, particularly emails emphasizing legal requirements for PDMP use. Such a low-cost, scalable email intervention may be of interest to policymakers and health care organizations seeking to promote PDMP use. Although these emails did not trigger large-scale changes in prescribing, similar interventions may be useful for promoting other best practices.

**Trial Registration:**

ClinicalTrials.gov Identifier: NCT06443385

## Key Points

**Question:**

Can reminder emails to clinicians increase Prescription Drug Monitoring Program (PDMP) use and encourage safer prescribing of opioids and other controlled substances?

**Finding:**

In this randomized clinical trial including 7872 clinicians, reminder emails significantly raised PDMP engagement, including account creation, database searches, and searches for patients with a history of risky prescribing. The effects persisted for at least 7 months and were approximately twice as large for emails that focused on the legal requirements mandating use of the PDMP rather than on its clinical benefits; prescribing quality and volume remained unchanged.

**Meaning:**

Email communications provide a low-cost, scalable approach to quickly increase PDMP use and assist clinicians seeking to prescribe medications safely, but the impact of reminder emails may not translate into large-scale changes in prescribing practices.

## Teaser

This randomized clinical trial investigates whether email communications increase clinician use of Prescription Drug Monitoring Programs to reduce guideline-discordant prescribing.

## Introduction

The opioid overdose death rate is falling rapidly in the US.^1^ Despite this progress, opioid prescribing and deaths from opioid prescriptions remain high compared with the start of the opioid overdose epidemic and relative to other high-income countries.^2,3,4^ Furthermore, prescribing of other risky drugs such as stimulants has grown and overdose deaths involving stimulants have risen exponentially.^4,5^

Prescription drug monitoring programs (PDMPs)—databases that track controlled substance prescription dispensing—offer clinicians a tool to ensure safer prescribing. By collecting and sharing data, PDMPs enable clinicians to check for potential drug interactions and verify that an intended prescription is safe to provide to the patient along with other recently dispensed medications. Guidelines from the US Centers for Disease Control and Prevention, Department of Veterans Affairs/Department of Defense, and the American Society of Interventional Pain Physicians recommend clinicians check a PDMP before writing new opioid prescriptions for pain.^6,7,8^ All states have operational PDMPs and the vast majority have a mandate requiring every prescriber of controlled substances to have an account and to check the database before prescribing an opioid.^9^

A growing body of evidence demonstrates that PDMP mandates make prescribing safer, likely by promoting greater use of these databases by clinicians.^10,11,12,13,14,15^ However, many clinicians still do not search PDMPs or even have accounts that would allow them to do so.^16^ In Minnesota, the setting for this trial, 32% of opioid prescriptions in 2023 came from clinicians who did not search the state’s PDMP; 20% of these clinicians did not even have a PDMP account.^17^

These statistics highlight an opportunity to further increase PDMP use, potentially improve prescribing safety, and promote guideline-concordant practices more generally. In a prior study,^18^ we found that mailed letters without protected health information increased PDMP account creation and search activity. We hypothesized that similar messages could be sent via email to increase PDMP use and improve prescribing safety. For example, work in a large health system demonstrated that emails to surgeons about prescribing guidelines durably increased guideline-concordant opioid prescribing after surgery.^19,20^ However, evidence on the feasibility and effects of email campaigns involving clinicians practicing in many health systems is limited.

This randomized clinical trial (intent-to-treat) sought to test whether email messages sent to Minnesota controlled-substance prescribers would increase PDMP engagement and encourage safer prescribing. Given the low cost and scalability of email communications, if they prove effective, they would make attractive tools for promoting PDMP use and improving the safety of prescribing.

## Methods

 This study was approved and overseen by the Columbia University institutional review board; it was exempt from informed consent requirements. The trial protocol is available in Supplement 1. This study followed the Consolidated Standards of Reporting Trials (CONSORT) reporting guideline.

### Study Design and Participants

The study used a parallel-group design with balanced randomization (1:1:1 ratio) of clinicians to a control group or 1 of 2 email intervention groups. In 1 intervention group, clinicians were sent emails emphasizing the state’s legal requirements to use the PDMP; in the other, clinicians were sent otherwise identical emails highlighting the clinical benefits of the PDMP. Study participants were physicians and physician assistants licensed in Minnesota who had not followed the state’s requirements to hold an active PDMP account or to check the PDMP before prescribing most opioids. The study was conducted from January 2024 to November 2025. Participants fell into 4 categories: those who prescribed controlled substances but (1) had no account or (2) had an inactive account; or those who prescribed opioids but (3) had never searched the PDMP or (4) searched infrequently relative to their opioid volume (ratio of searches to opioid dispenses ≤ 1:3). PDMP accounts are inactivated if clinicians fail to complete a required annual account review and accounts remain unusable until reactivated. To identify participants, we used state licensure and PDMP data, tracked prescribing during the 60-day period ending on June 30, 2024, and tracked account holding and searching during that period and additionally through July 15, 2024.

Prior to the trial, we used pretrial PDMP data to assess statistical power. We estimated that the study would have a minimum detectable effect of 2.8 percentage points for group vs group comparisons on a primary end point of increased PDMP engagement at 80% power and a 5% significance level.

### Intervention

Participants in both intervention groups were sent emails from the Minnesota PDMP bearing the logo of the state Board of Pharmacy. Emails began with a message about the clinician’s lack of engagement with the PDMP (this portion did not differ between the intervention groups). For instance, for clinicians without an account, the message noted that the clinician had prescribed controlled substances but did not have a PDMP account. Emails in the legal mandate group then described the Minnesota statute requiring account holding or searching. Emails in the clinical benefit group referenced the Centers for Disease Control and Prevention clinical practice guidelines^6^ and noted that account holding or searching would help clinicians follow these guidelines. Emails to both intervention groups provided clickable hyperlinks to create a PDMP account, reactivate an account, or search the PDMP, depending on the reason the clinician was enrolled in the study.

Initial emails were sent on July 17, 2024. Follow-up emails were sent on August 21, 2024, to the clinicians who had still not engaged with the PDMP as noted in the first email (created an account, reactivated an account, or searched, depending on the reason for study entry).

### Randomization

We used a stratified group randomization and computer-generated random numbers to assign clinicians to study groups. To maximize statistical power for guideline-discordant prescribing, we created strata of clinicians with similar anticipated values of this outcome.^21^ Specifically, using historical PDMP data, we trained a forecast model of a composite measure of guideline-discordant prescribing and then generated forecast values for study participants. Clinicians were stratified by enrollment reason (eg, no PDMP account), then sorted within strata by the forecasted composite prescribing measure. Groups of 3 adjacent clinicians were then assigned to strata for randomization.

### Data Sources

We obtained data from the Minnesota PDMP, which contains all of the controlled substance prescriptions dispensed within the state, all PDMP accounts, and all PDMP search queries. The data tracked prescribing and PDMP use through 7 months from the first email. We also used licensure data from the Minnesota Board of Medical Practice.

### Primary and Secondary Outcomes

We prespecified 2 primary end points, measured during the 60-day period after the first email. The first assessed PDMP use and was defined as an indicator for whether the clinician had engaged in the PDMP activity that triggered their study enrollment: account creation (for those who were enrolled because they lacked accounts), account activation (for those with inactive accounts), any PDMP search (for those who had not searched), or an increase in the search rate (for those who had searched infrequently, defined as an increase in the search query to opioid dispense ratio relative to the ratio at study enrollment).

The second primary end point assessed prescribing and was a composite of 5 measures of potentially guideline-discordant opioid prescribing: opioid-opioid overlap days involving multiple prescribers, opioid-benzodiazepine overlap days, opioid-gabapentinoid overlap days, days with high opioid doses (>90 morphine milligram equivalents), and dispenses with more than a 7-day supply to patients without prior opioid prescriptions. The measures were standardized to have zero mean and unit variance in the full sample and were averaged together. We prespecified secondary outcomes in 3 domains: email engagement (ie, open and click-through), PDMP use (ie, account creation, database searches, and the characteristics of searched patients), and prescribing (ie, the 5 guideline-discordant measures and prescribing volume in days supplied).

### Statistical Analysis

We used multivariable linear regressions to evaluate effects on study outcomes. To raise statistical power, we adjusted for the outcome as measured during the 60-day period immediately prior to the first emails as well as randomization strata. We used robust variance estimators for inference. Hypothesis tests were 2-sided and *P* < .05 was considered significant. To address the multiplicity of primary end points, we used the Westfall-Young step-down procedure to adjust these *P* values.^22^ Secondary outcomes were considered exploratory and were not adjusted for multiple testing. Statistical analyses were conducted from September 2024 to September 2025 using Stata, version 18 (StataCorp).

## Results

**Figure 1.**
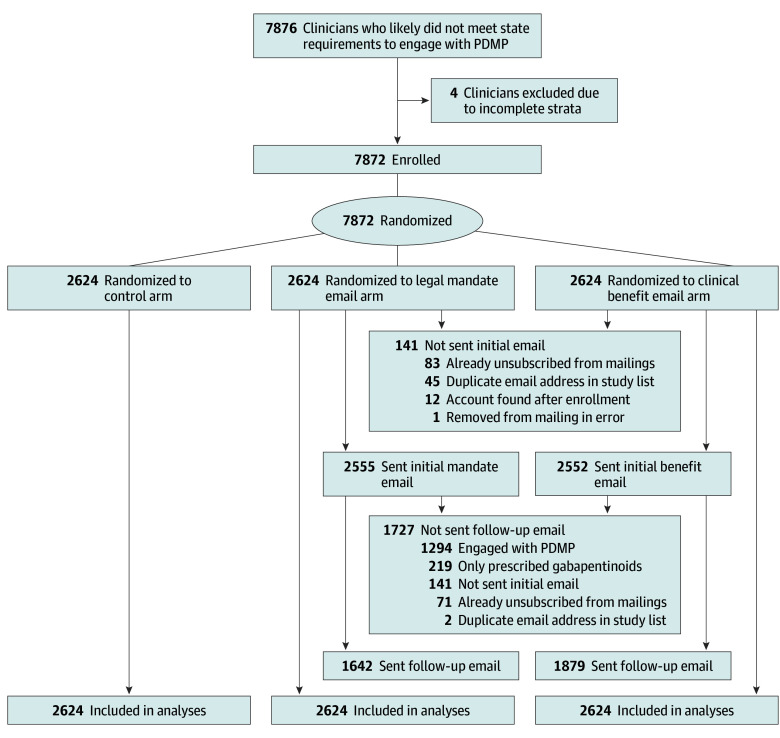
CONSORT Flow Diagram of Study Participants PDMP indicates the Prescription Drug Monitoring Program in Minnesota.

Of 7876 clinicians who met eligibility criteria, 4 were excluded due to incomplete strata; the remaining 7872 were enrolled and randomized evenly across study groups (2646 per group) and included in the analyses (Figure 1). Physicians comprised 6574 (83.5%) of study participants and physician assistants accounted for the remaining 1298 (16.5%); 4385 (55.7%) were men and 3487 (44.3%) were women (Table 1). Of the 5248 clinicians in the 2 intervention groups, 141 (2.7%) were not sent email messages, primarily because they had previously unsubscribed from PDMP email communications or had duplicate email addresses in the enrollment list; 5107 clinicians (97.3%) were sent emails. For follow-up emails, 1727 clinicians (32.9%) were excluded, either because they had taken the recommended PDMP action (eg, those who created accounts who initially lacked them); had unsubscribed from email communications; had been previously excluded; or had prescribed only gabapentinoids, which are controlled substances at the Minnesota state level but not at the national level (the final exclusion was added at the request of the PDMP).

**Table 1.** Characteristics of Participants, by Study Group

Characteristic	Group, No. (%)		
	Control	Email type	
		Legal mandate	Clinical benefit
Clinicians, No.	2624	2624	2624
Female	1148 (43.8)	1176 (44.8)	1163 (44.3)
Male	1476 (56.3)	1448 (55.2)	1461 (55.7)
Reason for study entry			
No account	1356 (51.7)	1356 (51.7)	1356 (51.7)
Inactive account	256 (9.8)	256 (9.8)	256 (9.8)
No search	585 (22.3)	585 (22.3)	585 (22.3)
Infrequent search	427 (16.3)	427 (16.3)	427 (16.3)
Clinician specialty			
Physician	2192 (83.5)	2202 (83.9)	2180 (83.1)
Internal medicine/primary care	866 (33.0)	815 (31.1)	832 (31.7)
Surgery	319 (12.2)	342 (13.0)	303 (11.5)
Physician assistant	432 (16.5)	422 (16.1)	444 (16.9)
PDMP use during baseline period^a^			
Had account	1269 (48.4)	1274 (48.6)	1270 (48.4)
Had active account	1012 (38.6)	1012 (38.6)	1012 (38.6)
Searched	342 (13.0)	357 (13.6)	318 (12.1)
Search count, mean (SD)	0.6 (3.5)	0.6 (2.5)	0.6 (2.7)
Guideline-discordant prescribing at baseline,^b^ mean (SD)			
Opioid-opioid overlap days	4.7 (18.7)	5.0 (24.0)	5.1 (25.0)
Opioid-benzodiazepine overlap days	1.4 (7.7)	1.3 (6.5)	1.4 (9.0)
Opioid-gabapentinoid overlap days	2.2 (5.3)	2.2 (5.1)	2.0 (4.9)
Patient-days with MEDD >90	2.1 (17.8)	2.3 (17.0)	2.9 (41.9)
Dispensing >7 d to patients new to opioids	0.7 (2.4)	0.7 (3.8)	0.8 (3.7)
Days supplied during baseline period, mean (SD)			
All controlled substances	732.5 (2128.7)	644.7 (1753.1)	731.0 (2488.8)
Opioids	109.4 (401.5)	107.2 (360.0)	102.0 (371.8)

Abbreviations: MEDD, morphine equivalent daily dose; PDMP, Prescription Drug Monitoring Program.

^a^ Because the baseline period included dates after clinicians were assessed for study entry, rates of PDMP engagement shown here do not necessarily match rates at study entry shown at the top of this table.

^b^ Number of clinicians in study group (share of clinicians in study group). Baseline period is the 60-day period immediately prior to the first emails being sent.

Among participants, 4068 (51.7%) were enrolled because they lacked a PDMP account, 768 (9.8%) because their account was inactive, 1755 (22.3%) because they did not search the PDMP, and 1281 (16.3%) because they searched infrequently. Participants prescribed an average of 702.7 days of controlled substances and 106.2 days of opioids during the 60-day period prior to the initial emails (Table 1).

### PDMP Engagement

**Figure 2.**
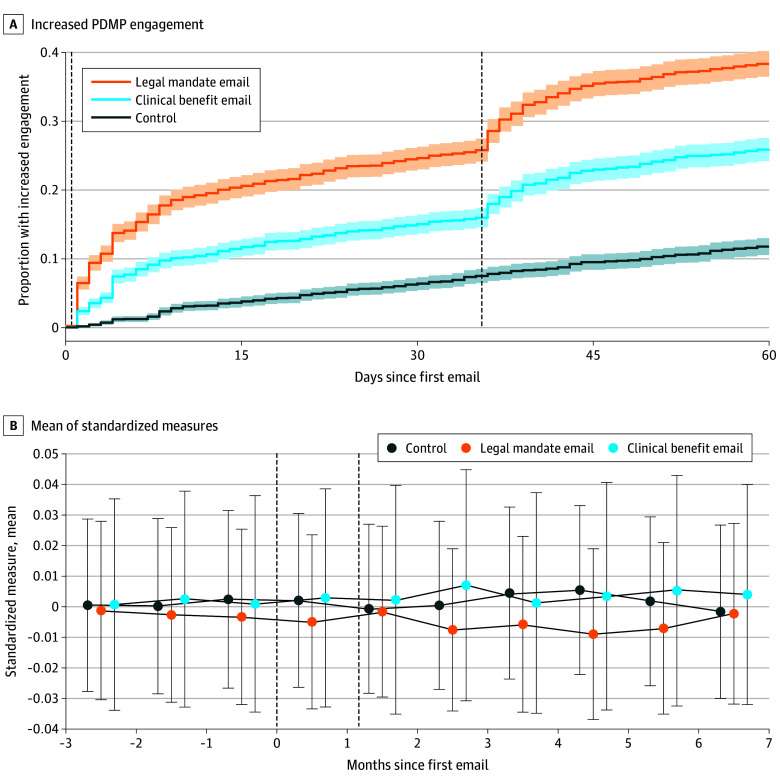
Prescription Drug Monitoring Program (PDMP) Engagement and Guideline-Discordant Opioid Prescribing by Study Group A, Depicts the PDMP engagement primary end point during the 60-day primary outcome period, with the lines showing the share of clinicians in each group that engaged with the PDMP by that day relative to the start of the intervention. The engagement measure is tailored to the reason the clinician was initially enrolled in the study: account creation for those enrolled because they lacked an account, account activation for those with an inactive account, initiating a search query for those who did not search, and increasing their ratio of searches to opioid dispenses for those who searched infrequently. B, Depicts the guideline-discordant opioid prescribing primary end point, which is the average of 5 standardized (mean [SD], 0 [1]) measures described in the text. Each point is the average among clinicians in the given month relative to the start of the intervention. The primary outcome period is from month 1 through 2 (60 days). The 95% CIs are represented by shading in panel A and whiskers in panel B; vertical dashed lines denote when emails were sent.

 As shown in Figure 2 and Table 2, during the 60-day postintervention period, 309 clinicians (11.8%) in the control group engaged with the PDMP, addressing the issue that qualified them for the study (eg, creating or activating an account, searching the PDMP, or searching it more frequently); in the legal mandate group, 1006 clinicians (38.3%) engaged with the PDMP, while in the clinical benefit group, 680 clinicians (25.9%) did so. The legal mandate email increased this rate by 26.5 (95% CI, 24.3-28.7; *P* < .001) percentage points while the clinical benefit email increased it by 14.2 (95% CI, 12.0-16.3; *P* < .001) percentage points.

**Table 2.** Effect of Interventions on Primary Outcomes and Selected Secondary Outcomes^a^

Outcome	Control mean, %	Email groups vs control			
		Legal mandate (n = 2624)		Clinical benefit (n = 2624)	
		Adjusted difference (95% CI)	*P* value	Adjusted difference (95% CI)	*P* value
Primary outcomes					
Increased PDMP engagement^b^	11.8	26.5 (24.3 to 28.7)	<.001^c^	14.2 (12.0 to 16.3)	<.001^c^
Guideline-discordant prescribing^d^	−0.001	0.001 (−0.010 to 0.012)	.98^c^	0.001 (−0.011 to 0.013)	.98^c^
Email delivery and email engagement outcomes					
Email delivered	0	94.7 (93.8 to 95.6)	<.001	94.9 (94.0 to 95.8)	<.001
Email opened	0	62.3 (60.3 to 64.3)	<.001	59.3 (57.3 to 61.3)	<.001
User clicked link in email	0	34.8 (32.9 to 36.6)	<.001	21.0 (19.2 to 22.7)	<.001
Selected PDMP engagement outcomes^e^					
Has account					
All participants	49.7	17.4 (15.9 to 18.9)	<.001	7.9 (6.6 to 9.2)	<.001
Enrolled due to no account	2.6	33.7 (31.0 to 36.4)	<.001	15.3 (12.8 to 17.8)	<.001
Active account					
All participants	40.1	21.1 (19.4 to 22.7)	<.001	10.8 (9.3 to 12.4)	<.001
Enrolled due to inactive account	<1.4^f^	36.3 (29.5 to 43.2)	<.001	30.9 (24.1 to 37.7)	<.001
Any search					
All participants	14.6	9.8 (8.1 to 11.5)	<.001	5.3 (3.6 to 7.0)	<.001
Enrolled due to no search	17.1	14.6 (9.8 to 19.5)	<.001	8.8 (4.0 to 13.6)	<.001
Met search target^g^					
All participants	11.1	9.6 (7.8 to 11.4)	<.001	5.4 (3.6 to 7.1)	<.001
Enrolled due to infrequent search	38.4	13.6 (7.0 to 20.2)	<.001	8.0 (1.3 to 14.6)	.02
Searches, No.	0.92	0.58 (0.38 to 0.79)	<.001	0.31 (0.12 to 0.49)	.001
Selected prescribing volume outcomes, days supplied, No.					
All controlled substances	737.83	−7.55 (−23.51 to 8.40)	.35	8.14 (−8.43 to 24.71)	.34
Opioids	109.02	0.39 (−3.82 to 4.60)	.86	2.62 (−1.95 to 7.18)	.26

Abbreviation: PDMP, prescription drug monitoring program.

^a^ Outcomes measure PDMP engagement and prescribing during the 60 days beginning when the first email was sent. Email delivery and engagement were measured during the 6 to 7 days after each email was sent. Adjusted differences are least squares differences between the specified treatment group and the control group, adjusted for the outcome as measured during the 60-day baseline period and the randomization strata.

^b^ Engagement measure differs by entry group: for clinicians enrolled because they lacked an account, the outcome was account creation; for inactive accounts, an active account; for those who did not search, any search; and those who searched infrequently, an indicator for an increase in their search rate (see footnote e).

^c^ *P* values adjusted for multiple testing using Westfall-Young stepdown procedure.

^d^ Outcome was the average of 5 standardized (mean [SD], 0 [1]) measures of guideline-discordant opioid prescribing: opioid-opioid overlap days, opioid-benzodiazepine overlap days, opioid-gabapentinoid overlap days, patient-days with morphine equivalent daily dose >90, and opioid dispensing with >7 days supply to patients new to opioids.

^e^ First 4 PDMP engagement outcomes are shown for the full study (all participants, the first indented row) and by the subgroup of the study for which the outcome was the primary PDMP engagement outcome (enrollment reason, the second indented row).

^f^ Exact value suppressed for privacy.

^g^ Indicator for ratio of searches to opioid dispensations during the outcome period greater than this ratio during the 60-day period immediately prior to study entry. If the clinician had no opioid dispensations during either period, this outcome was an indicator for any search.

The interventions significantly increased targeted PDMP engagement activity within each study entry group, increasing account holding for clinicians who were enrolled because they lacked accounts; active account holding for those enrolled because their accounts were inactive; searching for those enrolled because they did not search; and the search rate for those enrolled because they searched infrequently (eFigure 1 in Supplement 2; Table 2). Effect estimates were significantly larger for legal mandate emails than for clinical benefit emails (eTable 1 in Supplement 2).

### Guideline-Discordant Prescribing

The mean (SD) of the composite guideline-discordant prescribing measure was −0.001 (0.703) in the control group. The emails had no detectable effects on this outcome: for the legal mandate email, the adjusted difference was 0.001 (95% CI, −0.010 to 0.012; *P* = .98) units; and for the clinical benefit email, 0.001 (95% CI, −0.011 to 0.013; *P* = .98) units (Figure 2 and Table 2). Consistent with this null, all estimates of the 5 component measures were null except for opioid-benzodiazepine overlap days in the legal mandate email group, with an adjusted difference of −0.95 (95% CI, −1.90 to 0; *P* = .05) days (eFigure 2 and eTable 2 in Supplement 2).

### Secondary Outcomes

We measured email delivery, opening, and clicks (Table 2) to assess intervention fidelity. In the mandate email group, emails were delivered to 2485 participants (94.7%; 95% CI, 93.8-95.6), at least 1636 (62.3%; 95% CI, 60.3-64.3) opened the email (a lower bound estimate because the opening of an email can be tracked in some but not all email clients), and 912 (34.8%; 95% CI, 32.9-36.6) clicked at least 1 link. Email engagement was comparable in the clinical benefit email group, except the click-through rate was lower at 21.0% (95% CI, 19.2-22.7). All effects were significantly different from zero.

**Figure 3.**
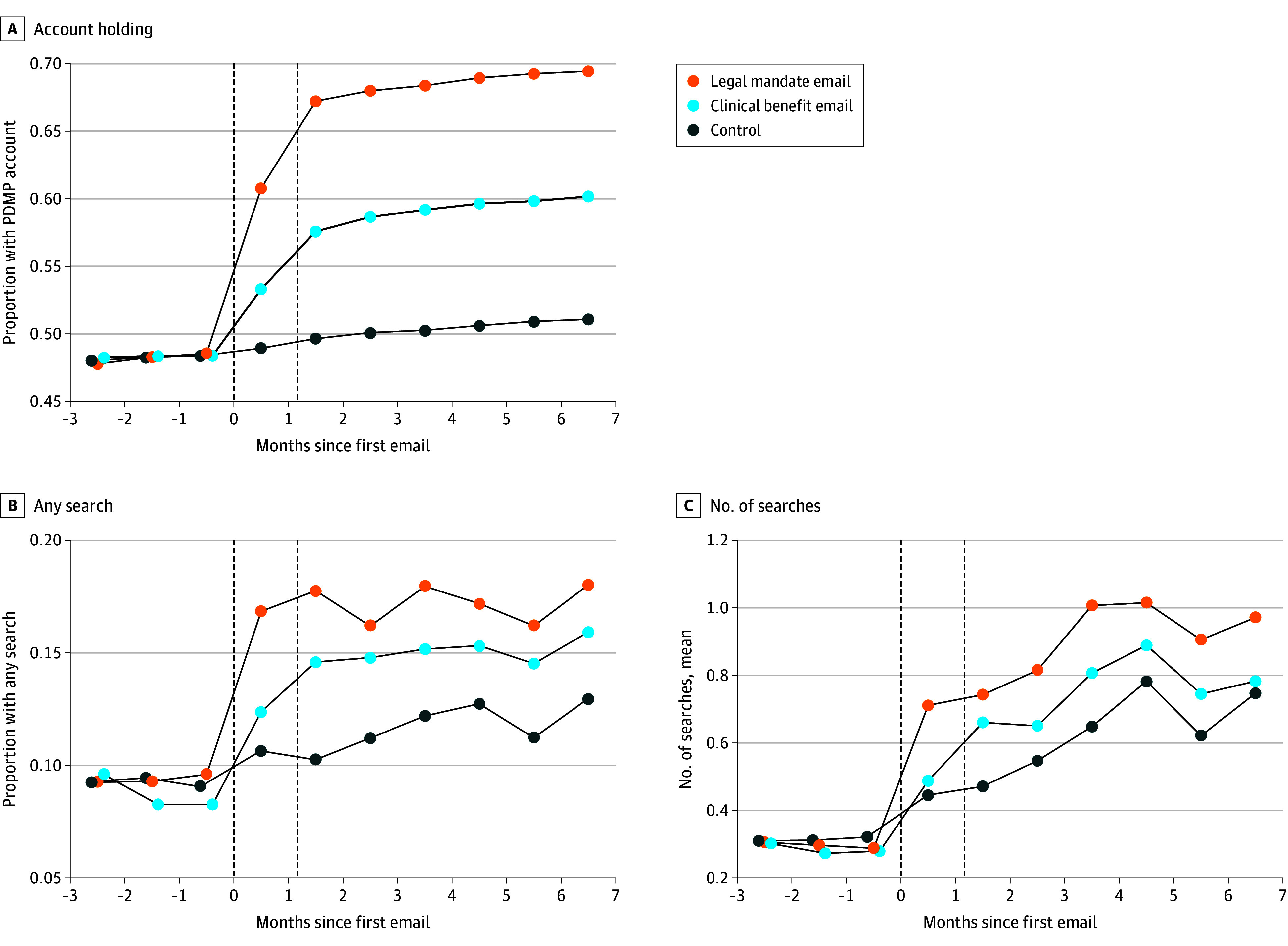
Prescription Drug Monitoring Program (PDMP) Account Holding and Searching Over Time Each panel depicts the evolution of a measure of PDMP engagement by study group, starting 3 months before and ending 7 months after the first emails were sent. Each point is the share among clinicians in the given study group who held a PDMP account or searched the PDMP in the given month (A and B, respectively) or the mean number of search queries among clinicians in the group in that month (C). eTable 7 in Supplement 2 provides estimates of the effects in each month. Vertical dashed lines denote when emails were sent.

We also considered a host of direct measures of PDMP engagement (Figure 3 and Table 2). At 60 days postintervention, 1303 clinicians (49.7%) in the control group had a PDMP account. The legal mandate email raised account holding by 17.4 (95% CI, 15.9-18.9; *P* < .001) percentage points and the clinical benefit email raised it by 7.9 (95% CI, 6.6-9.2; *P* < .001) percentage points. Results were similar for the rate of holding active accounts.

During this period, 383 clinicians (14.6%) in the control group searched the PDMP. The legal mandate email increased the share with any search by 9.8 (95% CI, 8.1-11.5; *P* < .001) percentage points while the clinical benefit email increased this share by 5.3 (95% CI, 3.6 to 7.1*; P *< .001) percentage points. Results were similar for the share meeting the search target, which we defined as a rise in the clinician’s ratio of searches to opioid dispenses relative to this ratio at the time of enrollment. The emails increased searches for clinicians irrespective of their enrollment trigger, ie, for those who lacked accounts, had inactive accounts, did not search, or searched infrequently at baseline (eTable 3 in Supplement 2).

The legal mandate emails induced 0.58 (95% CI, 0.38-0.79*; P *< .001) searches and the clinical benefit emails induced 0.31 (95% CI, 0.12-0.49*; P *= .001) searches relative to the control group’s mean (SD) searches of 0.92 (5.42). Both email types increased searching for patients who had previously received controlled substances, opioids, or guideline-discordant opioids (eTable 4 in Supplement 2). For example, legal mandate emails induced 0.17 additional searches for patients with a history of guideline-discordant opioid receipt (adjusted difference vs control; 95% CI, 0.09 to 0.24; *P* < .001) while clinical benefit emails induced 0.14 such searches (95% CI, 0.06 to 0.21; P < .001) relative to the control group’s mean (SD) of 0.31 (2.00) searches for these patients.

Account holding and search rates remained higher for at least 7 months in both of the email groups, approximately 6 months after the second emails were sent (Figure 3). Results for PDMP engagement were similar when measured at durations as short as 1 month and as long as 7 months (eTables 5 and 6 in Supplement 2). Assessing PDMP use in just month 7 postintervention, both emails continued to significantly raise the share of clinicians with accounts and with any PDMP searches (eTable 7 in Supplement 2).

Results on the primary prescribing outcome were quantitatively similar and remained null when measuring it at alternative durations (eTable 5 in Supplement 2) as were effects on alternative constructions of the component measures (eTable 8 in Supplement 2). There were no detected effects on controlled substance prescribing volume overall nor across drug classes (opioids, benzodiazepines, gabapentinoids, stimulants, opioid use disorder medications; eTables 2 and 8 in Supplement 2).

Results were similar across prespecified subgroups (physician vs physician assistant, active vs no active PDMP account at baseline), with both emails increasing PDMP engagement and the legal mandate email having approximately twice the effect as the clinical benefit email (eTable 9 in Supplement 2). There were no detected effects on guideline-discordant prescribing across subgroups.

## Discussion

In this randomized clinical trial of reminder emails, both message formats—highlighting either the PDMP’s legal requirements or its clinical benefits—increased PDMP engagement. Effects were largest for the legal mandate email message, which increased PDMP engagement by 26.5 percentage points, implying that 1 in 4 clinicians engaged with the PDMP who otherwise would not have. The effects persisted for at least 7 months.

PDMP engagement increased by twice as much from emails emphasizing legal mandates vs clinical benefits of the PDMP. The effectiveness of the mandate message may derive from its comparatively strong language focused on legal requirements. While the state’s PDMP account holding and search requirements began in 2017 and 2021, respectively, publicity about them has been limited. Thus, the mandate email may have provided new information to some clinicians. For others, the emails may have increased the mandates’ salience and signaled possible enforcement.

Despite large increases in PDMP engagement, we found no detectable effects on prescribing. Pooling the intervention groups to raise power, we can rule out even small reductions in prescribing across the 14 measures reported in eTable 2 in Supplement 2 (mean [SD] lower bound of 95% CI divided by control group mean: −6.3% [3.4] percentage points). These null prescribing results occurred despite extensive engagement with the PDMP. The increase in new searches was comparable in magnitude to the number of patients receiving risky opioids, although an order of magnitude smaller than the total number of patients taking opioids (eTable 8 in Supplement 2). A priori, it is unclear whether such an increase should have been sufficient to change prescribing, or whether larger increases in searching would have achieved this.

Our null results contrast with evidence that must-access PDMPs reduce risky prescribing.^10,12,13,14,15^ However, these studies often assessed earlier periods when unsafe prescribing was more common, and clinicians in this study tended to prescribe low volumes of risky opioids. Given the shift to more conservative opioid prescribing practices, further increases in PDMP use may no longer translate to large-scale changes in prescribing. Moreover, prior effects may reflect earlier adopters of PDMPs; the late adopters in this study may be less responsive to the information in PDMPs. Given that creating a PDMP account and searching for patients is time-consuming, one perspective on this finding is that the emails imposed time costs on clinicians absent prescribing benefits. Streamlining PDMP access through integration with electronic medical records could reduce those time costs and increase searches, an approach backed by randomized evidence.^23^

Regardless, these results may be encouraging for policymakers and organizations seeking to promote patient safety. First, the emails led many clinicians to make or reactivate PDMP accounts. These clinicians gained the ability to easily query the PDMP, avoiding the need to configure an account when a patient at risk of adverse outcomes presents for treatment. Second, both emails led clinicians to search the PDMP for at least 7 months and many of the queries were for patients who had received risky prescriptions. The increased search may help clinicians confirm which patients are at risk of overdose, counsel them about safely taking medications, and encourage them to carry naloxone. The practice of searching may also prompt clinicians to more closely evaluate medication appropriateness and even provide confirmation that a patient who could benefit from an opioid is at low risk of overdose.^24^

Our findings align with a previous study^18^ from our research team showing that mailed letters with information about the mandate drove more PDMP engagement than clinically focused letters centered on risky opioid prescribing, and that neither letter changed prescribing. While participant characteristics differed between the studies, it is notable that emails drove larger relative effects on PDMP use and larger absolute effects on account creation and any searching at negligible cost.

The effectiveness of this intervention further suggests that email can be an effective tool for promoting best practices in other clinical contexts. Our results also highlight that the content of the email message matters, underscoring the value of rigorous testing of this content for behavior change.

### Limitations

Our study has several limitations. First, it includes physicians and physician assistants in only 1 state. Results may differ in other geographic areas or for other practitioners. Second, the measures of guideline-discordant prescribing likely capture some clinically appropriate prescribing and miss some risky prescribing. Third, we cannot observe other changes that clinicians may have made, including potential increases in patient counseling or naloxone prescribing. Fourth, the interventions theoretically may have influenced the control group, although such effects are not apparent in the time pattern of PDMP engagement among the control group (Figure 2). Lastly, the data capture prescription dispensation and not actual medication use.

## Conclusions

In this randomized clinical trial, reminder emails emphasizing either a legal mandate or clinical benefits had large and durable effects on PDMP engagement as measured by active PDMP account holding and searching. Effects were largest for emails focused on legal requirements. No meaningful effects on prescribing were observed.

These findings should be of interest to policymakers, organizations, and clinicians seeking evidence-based tools to promote PDMP use and other best practices. The interventions we studied were simple email messages containing no protected health information, making it straightforward to adapt them to other settings. Reminder emails, particularly those emphasizing legal requirements, offer a practical, scalable, and low-cost strategy for facilitating guideline-concordant practices in health care delivery.

## Notes

Supplement 1. Trial Protocol

Supplement 2. eFigure 1. Primary PDMP Engagement Endpoint by Reason for Enrollment eFigure 2. Components of Primary Prescribing Endpoint eTable 1. Effect of Legal Mandate Email vs. Clinical Guideline Email on Primary Outcomes and Selected Secondary Outcomes eTable 2. Effects on Guideline-Discordant Prescribing Measures and Prescribing Volume eTable 3. Effects on PDMP Engagement by Study Enrollment Group eTable 4. Effects on PDMP Search and Searching for Patients with Prescribing Histories eTable 5. Effects on Primary Endpoints at Alternative Durations eTable 6. Effects on PDMP Engagement at Alternative Durations eTable 7. Effects on PDMP Engagement, Month-by-Month eTable 8. Effects on Alternative Constructions of Prescribing Measures eTable 9. Effects on Primary Endpoints by Subgroups

Supplement 3. Data Sharing Statement
