# Supplementary material for: Prescription Drug Monitoring Program Reminder Emails, Program Use, and Prescribing: A Randomized Clinical Trial
Source: JAMA Health Forum. 2025 Dec 19;6(12):e255623. doi: 10.1001/jamahealthforum.2025.5623 (PMC12717620; doi:10.1001/jamahealthforum.2025.5623)
Supplement: Supplement 1. — Trial Protocol [file jamahealthforum-e255623-s001.pdf]

Effect of Prescription Drug Monitoring Program Reminder E-mails on Program Use and Prescribing: A Randomized Clinical Trial

Supplement 1: Study Protocol

Adam Sacarny, PhD; Tatyana Avilova, PhD; Ian Williamson, MBA; Weston Merrick, PhD; Mireille Jacobson, PhD

Table of Contents

Study Methodology **3**
Identification of Study Clinicians 3
Randomization 4
Intervention Procedures 5
Initial E-mails 6
Follow-Up E-mails 7
Reproductions of Interventions **9**
Exhibit S1. Initial E-Mail – Legal Mandate Arm 9
Exhibit S2. Initial E-Mail – Clinical Benefit Arm 10
Exhibit S3. Follow-Up E-Mail – Legal Mandate Arm 11
Exhibit S4. Follow-Up E-Mail – Clinical Benefit Arm 12
Analysis Plan **13**
Introduction 13
Analysis Overview 14
Effects on PDMP Engagement 15
Effects on Prescribing 15
Measuring Implementation 16
Heterogeneous Effects 16
References 16
Appendix 16

Study Methodology

In this section, we describe the identification and enrollment of clinicians into the study, their randomization to study arms, and the procedures for conducting the e-mail intervention.

Identification of Study Clinicians

We obtained data on the universe of Minnesota-licensed physicians and physician assistants from the Minnesota Board of Medical Practice. We limited to clinicians in this data with an e-mail address and a valid National Provider Identifier (NPI).[1] To restrict the sample to clinicians who prescribed controlled substances, we linked this list to prescription dispense records from the PDMP. Specifically, we required that the clinician have at least one dispense record filled from April 3, 2024 through June 30, 2024.

We further limited the sample to clinicians who were likely not following state requirements to engage with the PDMP. There were four possible paths to enter the study due to this restriction, which we refer to as the four study entry groups. Clinicians were recorded as entering through the first path for which they qualified. The first two entry groups reflected the state's requirement to maintain an active PDMP account:

1. No account: we failed to find an account in the PDMP accounts file matching the clinician's NPI or license number.
2. Inactive account: we were able to link the clinician to their PDMP account, however, this account was flagged as inactive.

The remaining two entry groups reflected the state's requirement to query the PDMP before issuing most opioid prescriptions:

3. No search: the clinician had an active account, prescribed at least one opioid, but there were no searches in the PDMP associated with that account.
4. Infrequent search: the clinician had an active account, prescribed at least one opioid, and searched the PDMP, but they searched infrequently relative to their volume of prescribing. We defined infrequent search as a ratio of search queries to opioid dispenses of 1/3 or below.

For these classifications, account activity was measured on July 15, 2024, search queries were measured from April 3, 2024 through July 15, 2024, and opioid dispenses were measured from April 3, 2024 through June 30, 2024.

---

[1] We used NPIs to index clinicians in this study. However, our source datasets often lacked NPIs or contained invalid NPIs. For instance, the state licensure file had only license numbers for physician assistants and the prescription dispense data sometimes only included the prescriber's Drug Enforcement Agency (DEA) code or state license number. We used crosswalk data from LexisNexis to fill in NPIs based on the other identifiers available in datasets. Additionally, to ascertain additional NPIs in the licensure file, we developed and used a license number to NPI crosswalk from PDMP account records.

There were 7,876 clinicians who met the above criteria and were candidates for enrollment.

Randomization

We then randomized clinicians to study arms using a matched triples approach. We drew on Bai's study of optimal paired randomization methods and adapted a feasible procedure described on pp. 3922.[1]

In this procedure, one develops a cohort of individuals who would have been enrolled in a trial had it been conducted earlier and flexibly estimates the relationship between these individuals' covariates and the study's outcome of interest. Then, one uses these estimates to predict the study outcome of interest for the actual trial's participants, for whom the covariates are observed but the trial outcome is not (since the participants have not yet even been randomized). The participants are ordered by this prediction and adjacent units become the matched pairs (or, in our case, matched triples).

We implemented the procedure as follows. First, we assembled a predictor cohort of clinicians who would have met the study criteria described above according to account-holding as of April 2, 2024 and searching and prescribing from January 4, 2024 through April 2, 2024. Since the predictor cohort was assembled using earlier data, it was possible to observe "outcomes" for it. There were 8,232 clinicians in the cohort.

As the pre-specified primary outcome was defined as a composite of 5 guideline-discordant prescribing measures, we used the dispense data to construct these measures during a "baseline" period (January 4, 2024–April 2, 2024) and "endline" period (April 3, 2024–June 30, 2024). We defined the outcome for the predictor cohort by constructing the 5 prescribing measures during the endline period, standardizing each to 0 mean and unit variance, then averaging them together. We defined the covariates for the predictor cohort as the 5 measures during the baseline period.

Next, we used linear regression to estimate the relationship between this outcome and all two-way interactions between the covariates. The model yielded an R^2 of 93.5%.

Returning to the set of 7,876 clinicians who met enrollment criteria, we constructed the same covariates for the actual study's baseline period (April 3, 2024–June 30, 2024).[2] We then used the model estimates and these covariates to predict the outcome for these clinicians. For each of the four entry groups, we ordered the participants by their predicted outcome and assigned each group of adjacent three clinicians to a stratum. We dropped strata with fewer than 3 members. Due to the approach we used to order clinicians, these incomplete strata contained participants with the lowest predicted primary outcome.

[2] In analyses of study outcomes, we follow the pre-specified analysis plan and construct baseline controls using a different baseline period that runs the 60 days immediately prior to the send date of the first e-mails. At the time of randomization, prescription dispenses after June 30, 2024 were not yet available.

The result was a set of 7,872 clinicians assigned to 2,624 strata, each containing 3 clinicians in the same entry group. We used a sequence of computer-generated random numbers to randomly assign clinicians in each stratum to the study arms: control, legal mandate e-mails, and clinical benefit e-mails.

Intervention Procedures

The interventions were e-mails sent to participants in the two e-mail arms. Participants were eligible to receive up to 2 rounds of e-mails. The content of the e-mails varied depending on the participant's study arm and entry group; with 2 interventional arms and 4 entry groups, there were 8 e-mail templates in each round. If available, we used the participant's e-mail address associated with their PDMP account. Otherwise, we took their e-mail address from the licensure data.

Of the 5,248 participants assigned to the interventional arms, 12 were excluded from e-mails because PDMP staff found their PDMP accounts after enrollment and randomization. One additional participant was excluded in error. The PDMP also identified 45 participants with duplicate e-mail addresses (i.e. more than 1 participant had the same e-mail address) and excluded them. This step was necessary because the e-mail platform could not distinguish recipients with the same e-mail address. It also avoided the possibility of sending multiple e-mails to the same address.

The remaining participants were uploaded to the PDMP's e-mail delivery platform. The platform further excluded 83 participants who had previously unsubscribed from PDMP e-mails. On July 17, 2024, the remaining 5,107 participants (2,555 in the legal mandate arm, 2,552 in the clinical benefit arm) were sent initial e-mails.

We imposed additional restrictions on the second round of e-mails. Only participants sent an initial e-mail were eligible. We additionally removed the 1,294 participants who engaged with the PDMP in the manner described in the initial mailing. Specifically, for those in the "no account" group, we removed participants who created an account. For the "inactive account" group, we removed participants who had reactivated their account. Account creation and reactivation were measured as of August 19, 2024. For the "no search" and "infrequent search" groups, we removed participants with any search from July 17, 2024 through August 19, 2024.

After the original mailing, the PDMP reported receiving calls from clinicians who only prescribed gabapentinoids. Drugs in this class are controlled substances at the state but not federal level. Since clinicians could prescribe gabapentinoids without a DEA registration, it was not immediately certain if they were covered by the state account-holding mandate. To avoid causing any confusion with the second round of e-mails, we therefore removed an additional 219 clinicians whose only fills in the PDMP dispense data were gabapentinoids from April 3, 2024–July 31, 2024.

After removing an additional 2 participants with duplicate e-mails, the remaining participants were uploaded to the e-mail delivery platform. A further 71 participants were excluded because they had unsubscribed from e-mails. The second round of e-mails was sent on August 21, 2024

to the remaining 3,503 participants (1,624 in the legal mandate arm, 1,879 in the clinical benefit arm).

Initial E-mails

As previously noted, there were 8 templates for the initial e-mails (2 interventional arms and 4 entry groups). Exhibits S1 and S2 reproduce the templates for the e-mails sent to clinicians in the "low search" entry group.

The first paragraph of the e-mail differed by entry group, not by arm. The text was as follows:
- No account: "According to our records, you recently prescribed opioids or other controlled substances in Minnesota, but you do not have a Minnesota Prescription Monitoring Program (PMP) account.*"
- Inactive account: "According to our records, you recently prescribed opioids or other controlled substance in Minnesota, but your Minnesota Prescription Monitoring Program (PMP) account is inactive.*"
- No search: "According to our records, you recently prescribed opioids in Minnesota, but you haven't recently checked the Minnesota Prescription Monitoring Program (PMP).*"
- Infrequent search: "According to our records, you recently prescribed opioids in Minnesota, but you haven't regularly checked the Minnesota Prescription Monitoring Program (PMP).*"

The asterisked note came at the end of the e-mail and said:

> *Based on records as of July 13, 2024. Recent changes may not be reflected here."

The second paragraph differed by entry group and arm. In the legal mandate arm it read as follows:
- No account/inactive account: "Minnesota Statute §152.126 **requires** licensed clinicians practicing in Minnesota and authorized to prescribe controlled substances to register for and maintain a Minnesota PMP account."
- No search/infrequent search: "Minnesota Statute §152.126 **requires** prescribers, or delegates acting on their behalf, to check the PMP before issuing most opioid prescriptions initially and at least once every three months thereafter."

In the clinical benefit arm, the second paragraph always began:

> The 2022 CDC Clinical Practice Guideline recommends clinicians review PMP data "before every opioid prescription for acute, subacute, or chronic pain.

Then, a sentence tailored to the entry group followed:
- No account: "Registering for a PMP account will help you follow these recommendations and prescribe opioids safely."
- Inactive account: "Reactivating your PMP account will help you follow these recommendations and prescribe opioids safely."
- No search/infrequent search: "Checking the PMP will help you follow these recommendations and prescribe opioids safely."

The next section of the e-mail provided links for the recipient to engage in the proposed action. It differed by entry group, not by arm. It consisted of a heading, explanatory text, a clickable image with text, and a clickable text-only link so that recipients who did not load images could still see the link. The contents were as follows (heading / explanatory text / image and link text):

- No account: Registration is easy! / Click the link below to create a MN PMP account. / Create a MN PMP account
- Inactive account: Reactivating your PMP account is easy! / Click the link below to submit a secure request to program staff for assistance. / Request to reactivate my MN PMP account
- No search/infrequent search: Checking the PMP is easy! / Click the link below to access your MN PMP account. / Login to my MN PMP account

In the legal mandate arm only, we next included a clarification section. For the no account / inactive account entry groups, it read:

> More information about the registration requirement is available on our website located here. If you feel that your practice situation or license status may exempt you from the requirement to register for and maintain a PMP account, please contact a representative from your licensing board.

For the no search / infrequent search groups, it read:

> More information about the requirement to check the PMP, including the specific situations in which it applies, is available on our website here. This site includes a list of Frequently Asked Questions which may assist with unique Minnesota opiate prescribing situations.

Finally, the closing text was the same in all templates:

> This message is not intended to suggest how you should conduct your practice, but rather to offer information about uses of the PMP.
> Sincerely,
> Minnesota Prescription Monitoring Program

Follow-Up E-mails

There were also 8 follow-up email templates. The messages closely matched the original messages but were updated to acknowledge that the recipient had already been sent the notification. Exhibits S3 and S4 reproduce the templates for clinicians in the "low search" entry group.

The first paragraph was updated to read as follows:

- No account: "This message serves as a **reminder** of our previous communication dated July 17. The message stated that you recently prescribed opioids or other controlled substances in Minnesota, but you did not have a Minnesota Prescription Monitoring Program (PMP) account. According to our records, you still do not have an account.*"
- Inactive account: "This message serves as a **reminder** of our previous communication dated July 17. The message stated that you recently prescribed opioids or other controlled substance in Minnesota, but your Minnesota Prescription Monitoring Program (PMP) account was inactive. According to our records, your account is still inactive.*"

- No search: “This message serves as a **reminder** of our previous communication dated July 17. The message stated that you recently prescribed opioids in Minnesota, but you had not recently checked the Minnesota Prescription Monitoring Program (PMP). According to our records, you still have not checked the PMP.*”
- Infrequent search: “This message serves as a **reminder** of our previous communication dated July 17. The message stated that you recently prescribed opioids in Minnesota, but you had not regularly checked the Minnesota Prescription Monitoring Program (PMP). According to our records, you still have not regularly checked the PMP.*”

For clinicians in the no search / infrequent search entry groups, the message next stated:

> If you have not prescribed opioids since our last message, we recognize that you may not have had the opportunity to check the PMP yet.

The asterisked note was updated to read:

> * Based on records as of August 19, 2024. Recent changes may not be reflected here.

Reproductions of E-Mails

From: Minnesota PMP [redacted]@public.govdelivery.com
Subject: Important Message from the Minnesota Board of Pharmacy – Your Prescription Monitoring Program Use
Date: [redacted]
To: [redacted]

MP

Having trouble viewing this email? View it as a Web page.

MINNESOTA BOARD OF PHARMACY

Prescription Monitoring Program

Dear [redacted]

According to our records, you recently prescribed opioids in Minnesota, but you haven't regularly checked the Minnesota Prescription Monitoring Program (PMP).*

Minnesota Statute §152.126 **requires** prescribers, or delegates acting on their behalf, to check the PMP before issuing most opioid prescriptions initially and at least once every three months thereafter.

Checking the PMP is easy!

Click the link below to access your MN PMP account.

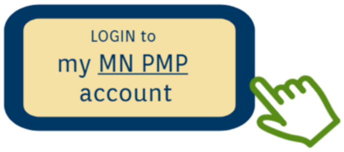

Login to my MN PMP account

More information about the requirement to check the PMP, including the specific situations in which it applies, is available on our website here. This site includes a list of Frequently Asked Questions which may assist with unique Minnesota opiate prescribing situations.

This message is not intended to suggest how you should conduct your practice, but rather to offer information about the use of the MN PMP.

Sincerely,

Minnesota Prescription Monitoring Program

---

* Based on records as of July 15, 2024. Recent changes may not be reflected here.

Please contact our program with questions.

ph. 651.201.2836 email. minnesota.pmp@state.mn.us web. mn.gov/boards/pharmacy-pmp

Stay Connected with the MN Board of Pharmacy

SUBSCRIBER SERVICES:
Manage Subscriptions | Unsubscribe All | Help

This email was sent to [redacted] using GovDelivery Communications Cloud, on behalf of: Minnesota Board of Pharmacy · 335 Randolph Ave., Suite 230 · St. Paul, MN 55102

Exhibit S1. Initial E-Mail – Legal Mandate Arm

From: Minnesota PMP [redacted]@public.govdelivery.com
Subject: Important Message from the Minnesota Board of Pharmacy – Your Prescription Monitoring Program Use
Date:
To: [redacted]

MP

Having trouble viewing this email? View it as a Web page.

MINNESOTA BOARD OF PHARMACY

Prescription Monitoring Program

Dear [redacted],

According to our records, you recently prescribed opioids in Minnesota, but you haven't regularly checked the Minnesota Prescription Monitoring Program (PMP).*

The 2022 CDC Clinical Practice Guideline recommends clinicians review PMP data "before every opioid prescription for acute, subacute, or chronic pain." Checking the PMP will help you follow these recommendations and prescribe opioids safely.

Checking the PMP is easy!

Click the link below to access your MN PMP account.

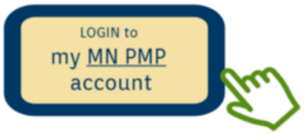

Login to my MN PMP account

This message is not intended to suggest how you should conduct your practice, but rather to offer information about uses of the MN PMP.

Sincerely,

Minnesota Prescription Monitoring Program

* Based on records as of July 15, 2024. Recent changes may not be reflected here.

Please contact our program with questions.

ph. 651.201.2836 email. minnesota.pmp@state.mn.us web. mn.gov/boards/pharmacy-pmp

Stay Connected with the MN Board of Pharmacy

SUBSCRIBER SERVICES:
Manage Subscriptions | Unsubscribe All | Help

This email was sent to [redacted] using GovDelivery Communications Cloud, on behalf of: Minnesota Board of Pharmacy · 335 Randolph Ave., Suite 230 · St. Paul, MN 55102

Exhibit S2. Initial E-Mail – Clinical Benefit Arm

From: Minnesota PMP [redacted]@public.govdelivery.com
Subject: REMINDER: Important Message from the Minnesota Board of Pharmacy – Your Prescription Monitoring Program Use
Date: [redacted]
To: [redacted]

MP

Having trouble viewing this email? View it as a Web page.

MINNESOTA BOARD OF PHARMACY

Prescription Monitoring Program

Dear [redacted]

This message serves as a **reminder** of our previous communication dated July 17. The message stated that you recently prescribed opioids in Minnesota, but you had not regularly checked the Minnesota Prescription Monitoring Program (PMP). According to our records, you still have not regularly checked the PMP.*

Minnesota Statute §152.126 **requires** prescribers, or delegates acting on their behalf, to check the PMP before issuing most opioid prescriptions initially and at least once every three months thereafter.

If you have not prescribed opioids since our last message, we recognize that you may not have had the opportunity to check the PMP yet.

Checking the PMP is easy!

Click the link below to access your MN PMP account.

LOGIN to
my MN PMP
account

Login to my MN PMP account

More information about the requirement to check the PMP, including the specific situations in which it applies, is available on our website here. This site includes a list of Frequently Asked Questions which may assist with unique Minnesota opiate prescribing situations.

This message is not intended to suggest how you should conduct your practice, but rather to offer information about the use of the MN PMP.

Sincerely,

Minnesota Prescription Monitoring Program

---

* Based on records as of August 19, 2024. Recent changes may not be reflected here.

Please contact our program with questions.

ph. 651.201.2836 email. minnesota.pmp@state.mn.us web. mn.gov/boards/pharmacy-pmp

Stay Connected with the MN Board of Pharmacy

SUBSCRIBER SERVICES:
Manage Subscriptions | Unsubscribe All | Help

This email was sent to [redacted] using GovDelivery Communications Cloud, on behalf of: Minnesota Board of Pharmacy · 335 Randolph Ave., Suite 230 · St. Paul, MN 55102

Exhibit S3. Follow-Up E-Mail – Legal Mandate Arm

From: Minnesota PMP [redacted]@public.govdelivery.com
Subject: REMINDER: Important Message from the Minnesota Board of Pharmacy – Your Prescription Monitoring Program Use
Date: [redacted]
To: [redacted]

MP

Having trouble viewing this email? View it as a Web page.

MINNESOTA BOARD OF PHARMACY

Prescription Monitoring Program

Dear [redacted]

This message serves as a **reminder** of our previous communication dated July 17. The message stated that you recently prescribed opioids in Minnesota, but you had not regularly checked the Minnesota Prescription Monitoring Program (PMP). According to our records, you still have not regularly checked the PMP.*

The 2022 CDC Clinical Practice Guideline recommends clinicians review PMP data "before every opioid prescription for acute, subacute, or chronic pain." Checking the PMP will help you follow these recommendations and prescribe opioids safely.

If you have not prescribed opioids since our last message, we recognize that you may not have had the opportunity to check the PMP yet.

Checking the PMP is easy!

Click the link below to access your MN PMP account.

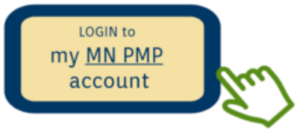

Login to my MN PMP account

This message is not intended to suggest how you should conduct your practice, but rather to offer information about uses of the MN PMP.

Sincerely,

Minnesota Prescription Monitoring Program

---

* Based on records as of August 19, 2024. Recent changes may not be reflected here.

Please contact our program with questions.

ph. 651.201.2836 email. minnesota.pmp@state.mn.us web. mn.gov/boards/pharmacy-pmp

Stay Connected with the MN Board of Pharmacy

SUBSCRIBER SERVICES:
Manage Subscriptions | Unsubscribe All | Help

This email was sent to [redacted] using GovDelivery Communications Cloud, on behalf of: Minnesota Board of Pharmacy · 335 Randolph Ave., Suite 230 · St. Paul, MN 55102

Exhibit S4. Follow-Up E-Mail – Clinical Benefit Arm

Analysis Plan

The following repeats the final archived version of the study analysis plan. This document was archived on the AEA RCT Registry on July 15, 2024, prior to the start of the interventions. It can be accessed there at: https://www.socialscienceregistry.org/trials/13549

The analysis plan proposes studying the average characteristics of patients searched by clinicians (Gruber et al., 1999). In practice, we used a simpler but closely related approach of reporting effects on the volume of searches for patients with various characteristics.

Abstract: Drug overdose deaths have skyrocketed in recent years, and many overdoses continue to involve prescribed medications like opioids and stimulants. At the same time, state prescription drug monitoring programs (PDMPs), which help clinicians prescribe these medications safely, remain underused. In Minnesota, 32% of opioid prescriptions are written by clinicians who do not use the PDMP. In many states, including Minnesota, policymakers have limited tools to raise PDMP use even though its use is often required under state law. To address this policy dilemma, we will test e-mails designed to encourage PDMP use and evaluate their effects on PDMP use and controlled substance prescribing. Our work will include a projected 7,126 physician and physician assistant prescribers of opioids and other controlled substances who lack active PDMP accounts, never query the PDMP, or query the PDMP infrequently relative to their prescribing volume. We will randomly assign these prescribers to be sent (1) emails focusing on the legal requirements to use the PDMP, (2) emails focusing on the clinical benefits of the PDMP or (3) no emails. Our work will generate evidence on low-cost approaches to encourage PDMP use and safer controlled substance prescribing.

Introduction

This document details an analysis plan for measuring the impact of emails sent to Minnesota physicians and physician assistants who are not following the state's requirements to engage with the state's prescription drug monitoring program (PDMP). At the time of writing, we have seen only the original pre-intervention search, account, and query data from the PDMP which was used to create a list of clinicians to deliver the intervention to. We may perform additional analyses in the course of the study in addition to those specified below; if analyses are not pre-specified in this document we will make note of that fact when sharing results.

Intervention

Clinicians are included in this study if they prescribe controlled substances but either lack a PDMP account or have an inactive account; prescribe opioids but do not search the PDMP at all; or prescribe opioids but search infrequently relative to their prescribing. Based on an analysis of 2023 PDMP data, we anticipate that 7,126 clinicians meet these criteria and will be enrolled in the study.

Clinicians will be randomized at 1:1:1 ratio to two treatment arms or an “as usual” control arm. One treatment arm will be sent e-mails highlighting the state’s legal requirements for prescribers to use the PDMP. The other treatment arm will be sent emails highlighting the clinical benefits

of having access to the PDMP, particularly before prescribing opioids. Both treatment arms are sent an initial email and a follow-up email one month later. Clinicians in the control arms will not be sent any emails from this experiment but, like clinicians in the treatment arms, may get other emails from the PDMP. The e-mails will be sent via the e-mail platform GovDelivery.

Primary outcome overview
The goal of this study is to understand whether e-mails to prescribers with low or no PDMP engagement can increase PDMP use and thereby encourage safer prescribing. This study's two **primary outcomes** measure PDMP engagement and rates of potentially guideline-discordant opioid prescribing. They are described later in the analysis plan. For hypothesis testing, we will use multiple testing adjustment to control the family-wise error rate to 5%.

Secondary outcome overview
Secondary outcomes will be measured both within 2-months of the initial email as well as at other intervals. We will also consider as secondary outcomes the components of our primary endpoints as well as our primary outcomes measured at other time intervals. These outcomes will be treated as exploratory and so we will not use multiple testing adjustments when reporting their p-values.

Data
The trial will use search, account, and prescribing data from the PDMP as well as e-mail data from GovDelivery.

Analysis Overview

Regressions
We begin by pooling the two intervention arms and estimating the regression model:

$$Y_i = \alpha + \beta \cdot EMAIL_i + \delta X_i + \gamma Z_i + \varepsilon_i , \quad (1)$$

where i indexes participants, Y_i is the outcome, $EMAIL_i$ is an indicator for being assigned to either interventional arm, X_i is a vector of controls, and Z_i is a vector of strata indicators. To raise statistical power, we will include in X_i the lagged (pre-e-mail) dependent variable and controls for specialization.

To distinguish between effects of each letter, we will also include indicators for each arm separately:

$$Y_i = \alpha + \beta_1 \cdot MANDATE_i + \beta_2 \cdot CLINICAL_i + \delta X_i + \gamma Z_i + \varepsilon_i . \quad (2)$$

Controls
The main controls we will use are lagged (pre-e-mail) dependent variables and fixed effects for provider specialization.

Strata
Using historical PDMP data, we estimate the relationship between baseline covariates and the primary prescribing outcome. When the actual list of clinicians in the trial is confirmed, we will use these estimates to predict each clinician's primary prescribing outcome and stratify on the

predictions in blocks of three. This stratification method was employed successfully in our previous trial.

Duration
Our primary outcomes are measured during the 2-month period after the first e-mail is sent.

We will also measure outcomes at shorter and longer durations. Specifically, to look for evidence on the timing and persistence of impacts, we will consider outcomes by month for up to nine months after emails are sent.

Effects on PDMP Engagement
The PDMP engagement primary outcome is an indicator of PDMP engagement during the 2-month period after the first e-mail is sent. This will indicate whether the clinician's level of engagement rose, relative to the criteria for inclusion in the study. The outcome will be defined as follows:

- Account creation for clinicians enrolled because they lacked one
- Reactivating an account for clinicians enrolled because their account was inactive
- Any search for clinicians enrolled because they never searched
- Increased search rates for clinicians enrolled because they infrequently searched[3]

Secondary outcomes related to engagement
Secondary outcomes related to PDMP engagement include the components of the primary endpoint measured separately (account creation, account reactivation, any search, and above-threshold search rate) as well as our primary outcome measured at alternative intervals, total search query volume, creation of delegate users,[4] and average characteristics of patients searched by clinicians, described below.

We construct the average characteristics of patients searched by the clinician to assess the targeting of PDMP searches. Average characteristics include prior prescription drug receipt and prior receipt of risky prescription medication interactions. We compare characteristics of the marginal patient searched due to the intervention compared to the infra-marginal patient (see Gruber et al., 1999).

Effects on Prescribing
The prescribing primary outcome is a composite measure of several guideline-discordant opioid prescribing behaviors within 2 months of email deployment. The components of this measure are:

- Opioid co-prescriptions with other opioids
- Co-prescriptions of opioids with benzodiazepines
- Co-prescriptions of opioids with gabapentinoids
- High daily opioid doses (morphine-equivalent daily dose >90)

---

[3] We define infrequent searching as a clinician whose search count is less than 1/3 their opioid fill count. Because clinicians can search before prescribing drugs other than opioids, this approach is, if anything, conservative.
[4] Delegate users are accounts that can check the PDMP on behalf of clinicians.

- Long-duration opioid prescriptions (>7 days) to opioid-naive individuals

The components will each be standardized to have mean 0 and standard deviation 1. Then, the standardized components will be averaged together to create the composite primary endpoint.

Secondary outcomes related to prescribing endpoints
In addition to guideline-discordant opioid prescribing, we measure prescribing volume, including:

- Total days supplied of controlled substances
- Total days supplied of opioids
- Total days supplied of benzodiazepines
- Total days supplied of gabapentinoids
- Total days supplied of stimulants
- Total opioid morphine milligram equivalents

We also include the components of our primary outcome measured separately as well as our primary outcome measured at alternative intervals.

Measuring Implementation
Measuring e-mail engagement assesses mechanisms driving PDMP engagement or guideline-discordant prescribing effects, or lack thereof. To understand the potential of our email communication, we will measure:

- Email open
- Click-through on e-mail links
- Email bounce

E-mail engagement is a secondary outcome and can be used to scale any effects to generate a "treatment on the treated" estimate.

Heterogeneous Effects
We will examine heterogeneity in effects across the following groups:

- Physicians (MD/DO) vs. physician assistants
- No active PDMP account at baseline vs. active PDMP account at baseline

References
Gruber, J., Levine, P., Staiger, D., 1999. Abortion Legalization and Child Living Circumstances: Who is the "Marginal Child"? Q. J. Econ. 114, 263–291. https://doi.org/10.1162/003355399556007

Appendix
Poisson Regression Specification
We may consider Poisson regressions because we are interested in percent changes in outcomes due to the emails. The Poisson regression will let us produce these statistics even if the outcome is sometimes zero.

The regressions will assume that the physician-level outcome takes the form:

$$y_i = exp\ (\alpha + \beta * TREAT_i + \delta X_i\ + \gamma Z_i) + e_i$$

The coefficient of interest in the Poisson regressions is β. This coefficient can be interpreted as the percent change in y due to the email, analogous to an OLS regression with ln (y) on the left-hand side.
