# Supplementary material for: Prescription Drug Monitoring Program Reminder Emails, Program Use, and Prescribing: A Randomized Clinical Trial
Source: JAMA Health Forum. 2025 Dec 19;6(12):e255623. doi: 10.1001/jamahealthforum.2025.5623 (PMC12717620; doi:10.1001/jamahealthforum.2025.5623)
Supplement: Supplement 2. — eFigure 1. Primary PDMP Engagement Endpoint by Reason for Enrollment eFigure 2. Components of Primary Prescribing Endpoint eTable 1. Effect of Legal Mandate Email vs. Clinical Guideline Email on Primary Outcomes and Selected Secondary Outcomes eTable 2. Effects on Guideline-Discordant Prescribing Measures and Prescribing Volume eTable 3. Effects on PDMP Engagement by Study Enrollment Group eTable 4. Effects on PDMP Search and Searching for Patients with Prescribing Histories eTable 5. Effects on Primary Endpoints at Alternative Durations eTable 6. Effects on PDMP Engagement at Alternative Durations eTable 7. Effects on PDMP Engagement, Month-by-Month eTable 8. Effects on Alternative Constructions of Prescribing Measures eTable 9. Effects on Primary Endpoints by Subgroups [file jamahealthforum-e255623-s002.pdf]

Supplemental Online Content

Sacarny A, Avilova T, Williamson I, Merrick W, Jacobson M. Prescription drug monitoring program reminder emails, program use, and prescribing: a randomized clinical trial. *JAMA Health Forum.* Published online December 19, 2025. doi:10.1001/jamahealthforum.2025.5623

eFigure 1. Primary PDMP Engagement Endpoint by Reason for Enrollment

eFigure 2. Components of Primary Prescribing Endpoint

eTable 1. Effect of Legal Mandate Email vs. Clinical Guideline Email on Primary Outcomes and Selected Secondary Outcomes

eTable 2. Effects on Guideline-Discordant Prescribing Measures and Prescribing Volume

eTable 3. Effects on PDMP Engagement by Study Enrollment Group

eTable 4. Effects on PDMP Search and Searching for Patients with Prescribing Histories

eTable 5. Effects on Primary Endpoints at Alternative Durations

eTable 6. Effects on PDMP Engagement at Alternative Durations

eTable 7. Effects on PDMP Engagement, Month-by-Month

eTable 8. Effects on Alternative Constructions of Prescribing Measures

eTable 9. Effects on Primary Endpoints by Subgroups

This supplemental material has been provided by the authors to give readers additional information about their work.

© 2025 Sacarny A et al. *JAMA Health Forum*

Table of Contents

eFigures **3**

eFigure 1: Primary PDMP Engagement Endpoint by Reason for Enrollment 3

eFigure 2: Components of Primary Prescribing Endpoint 4

eTables **5**

eTable 1: Effect of Legal Mandate E-mail vs. Clinical Guideline E-mail on Primary Outcomes and Selected Secondary Outcomes 5

eTable 2: Effects on Guideline-Discordant Prescribing Measures and Prescribing Volume 6

eTable 3: Effects on PDMP Engagement by Study Enrollment Group 7

eTable 4: Effects on PDMP Search and Searching for Patients with Prescribing Histories....... 8

eTable 5: Effects on Primary Endpoints at Alternative Durations 9

eTable 6: Effects on PDMP Engagement at Alternative Durations 10

eTable 7: Effects on PDMP Engagement, Month-by-Month 11

eTable 8: Effects on Alternative Constructions of Prescribing Measures 12

eTable 9: Effects on Primary Endpoints by Subgroups 13

© 2025 Sacarny A et al. *JAMA Health Forum*

eFigures

eFigure 1: Primary PDMP Engagement Endpoint by Reason for Enrollment

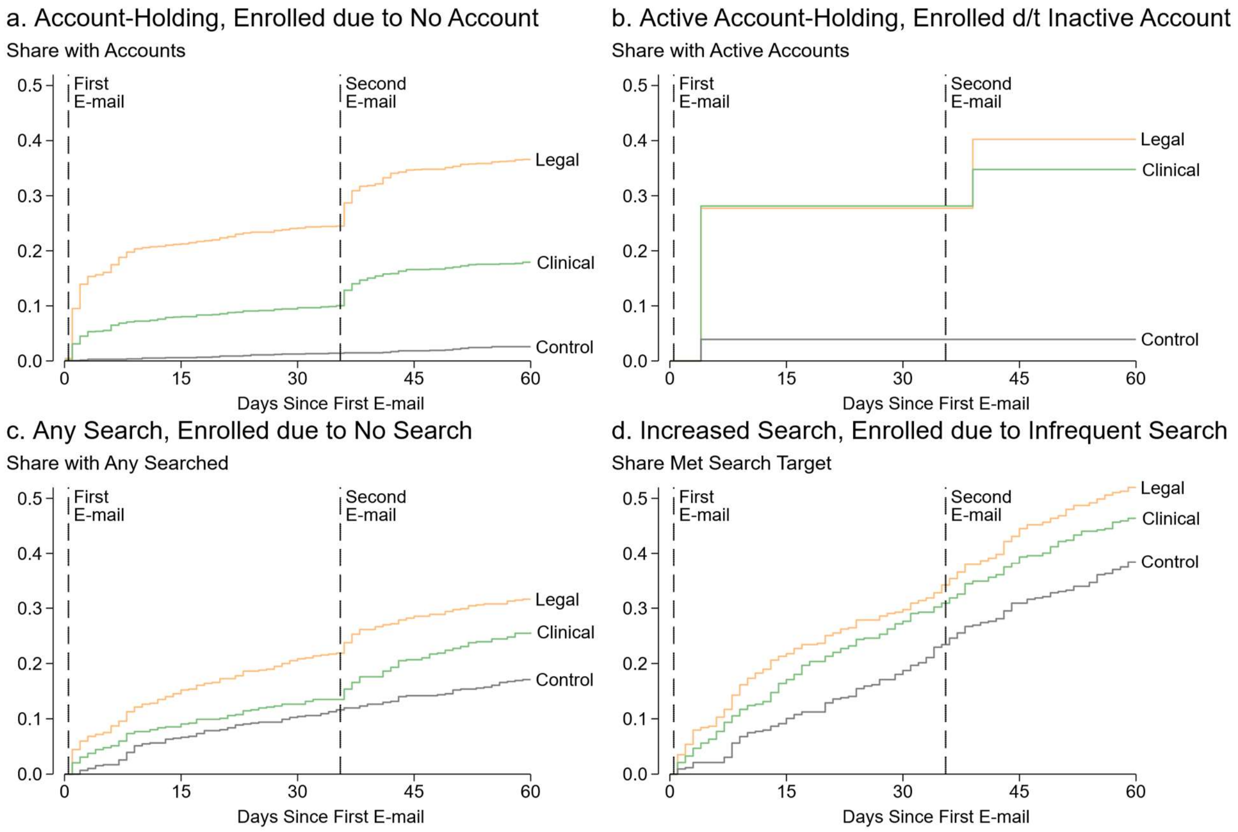

Caption: The primary PDMP engagement outcome (see Figure 2a in the main text) measures a different behavior depending on the participant's reason for enrollment. Each panel in this plot corresponds to a group enrolled for a different reason: no account, inactive account, no search, or infrequent search (in order from panel a to d). Each panel shows the share of clinicians in each arm that engaged with the PDMP according to the outcome measure described in the panel's title by that day relative to the start of the intervention. For instance, in panel a, the lines measure the share of participants with an account among those who were enrolled in the study because they lacked accounts.

© 2025 Sacarny A et al. *JAMA Health Forum*

eFigure 2: Components of Primary Prescribing Endpoint

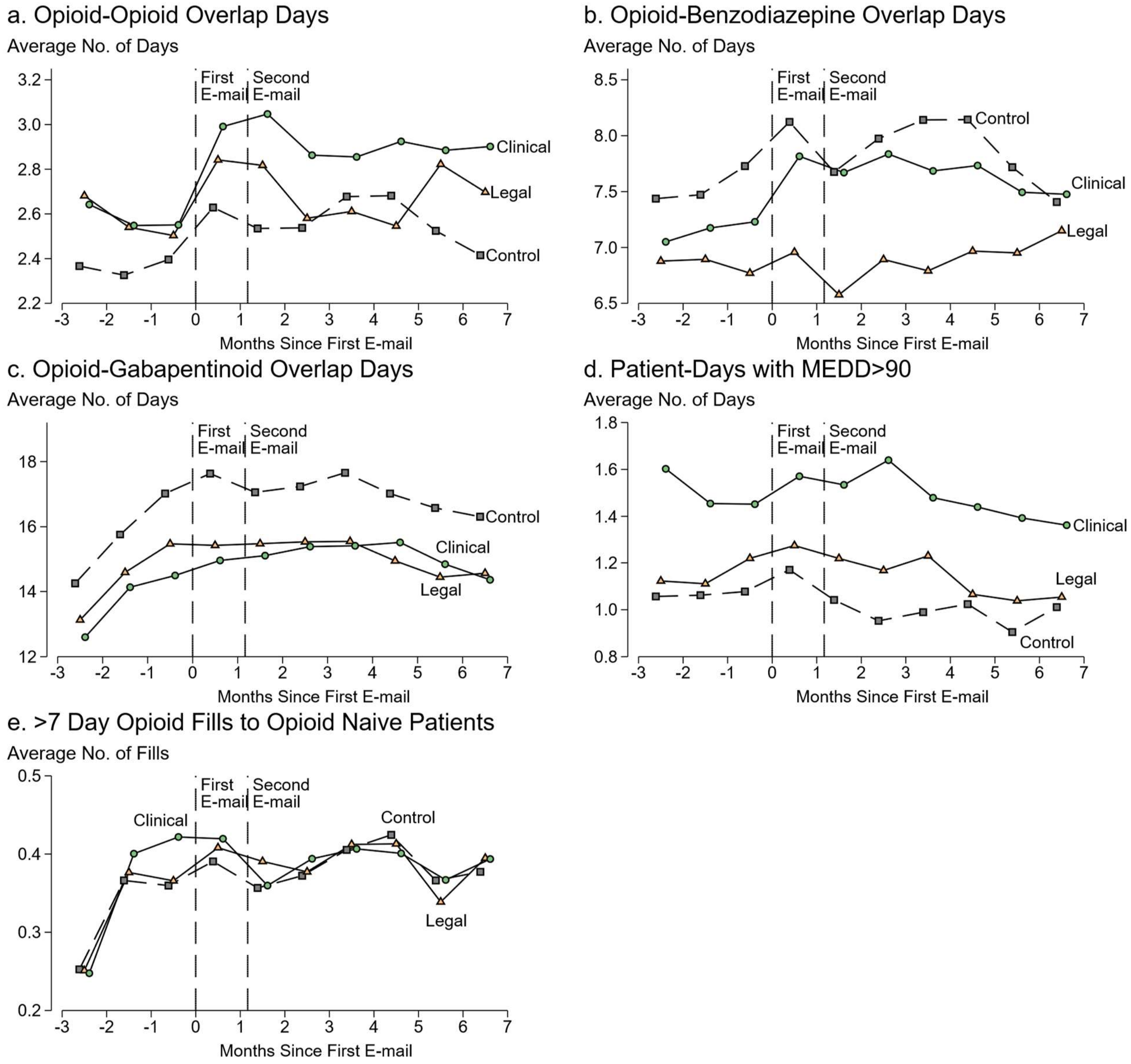

Caption: The primary prescribing outcome is a composite of 5 standardized measures of guideline-discordant opioid prescribing. Each panel in this plot pertains to a different prescribing measure and depicts the raw (un-standardized) measure month-by-month for each study arm. Each point is the average among clinicians in the given month relative to the start of the intervention. The primary outcome period is months 1 and 2 (60 days). Vertical dashed lines denote when e-mails were sent

© 2025 Sacarny A et al. *JAMA Health Forum*

eTables

eTable 1: Effect of Legal Mandate E-mail vs. Clinical Guideline E-mail on Primary Outcomes and Selected Secondary Outcomes

	Legal E-Mail Mean	Clinical E-mail Mean	Legal E-mail vs. Clinical E-mail Adj. Difference (95% CI)	*P* value
**Primary Outcomes**				
Increased PDMP Engagement, %	38.3%	25.9%	12.4 (10.0 to 14.7)	<0.001[a]
Guideline-Discordant Prescribing	-0.004	0.005	0.000 (-0.012 to 0.012)	0.99[a]
**E-mail Delivery and E-mail Engagement**				
E-mail Delivered, %	94.7%	94.9%	-0.2 (-1.3 to 0.9)	0.73
E-mail Opened, %	62.3%	59.3%	3.0 (0.7 to 5.4)	0.01
User Clicked Link in E-mail, %	34.8%	21.0%	13.8 (11.6 to 16.0)	<0.001
**PDMP Engagement**				
Has Account, %				
All Participants	67.2%	57.6%	9.5 (7.9 to 11.1)	<0.001
Enrolled d/t No Account	36.6%	17.9%	18.5 (15.4 to 21.5)	<0.001
Active Account, %				
All Participants	61.2%	50.9%	10.3 (8.5 to 12.0)	<0.001
Enrolled d/t Inactive Account	40.2%	34.8%	5.5 (-2.2 to 13.1)	0.16
Any Search, %				
All Participants	24.5%	19.7%	4.5 (2.7 to 6.3)	<0.001
Enrolled d/t No Search	31.6%	25.6%	5.9 (0.8 to 10.9)	0.02
Met Search Target, %				
All Participants	20.7%	16.5%	4.2 (2.4 to 6.1)	<0.001
Enrolled d/t Infrequent Search	52.0%	46.4%	5.6 (-1.1 to 12.4)	0.10
No. of Searches	1.45	1.15	0.27 (0.07 to 0.48)	0.008
**Prescribing Volume, Days Supplied**				
All Controlled Substances	641.98	744.47	-15.70 (-31.63 to 0.24)	0.05
Opioids	107.29	104.45	-2.23 (-6.92 to 2.47)	0.35

* This table repeats Table 2 but reports adjusted differences between the two e-mail arms. These are least squares differences between the legal mandate e-mail group and the clinical benefit e-mail group, adjusted for the outcome as measured during the 60-day baseline period and the randomization strata. See notes to Table 2 for more details.

a These *P* values differ from those reported in Table 2 because they are not adjusted for multiple testing. However, the first *P* value is less than 10^{-24}.

PDMP: Prescription Drug Monitoring Program

© 2025 Sacarny A et al. *JAMA Health Forum*

eTable 2: Effects on Guideline-Discordant Prescribing Measures and Prescribing Volume

	Control	Legal Mandate E-mail vs. Control		Clinical Benefit E-mail vs. Control	
	Mean	Adj. Difference (95% CI)	*P* value	Adj. Difference (95% CI)	*P* value
**Guideline-Discordant Prescribing Measures (Components of Primary Prescribing Endpoint)**					
Opioid-Opioid Overlap Days	4.85	0.14 (-0.50 to 0.78)	0.66	0.41 (-0.28 to 1.09)	0.24
Opioid-BZ Overlap Days	15.12	-0.95 (-1.90 to 0.00)	0.05	0.26 (-0.84 to 1.37)	0.64
Opioid-GB Overlap Days	33.98	-1.04 (-2.42 to 0.34)	0.14	-0.40 (-1.87 to 1.06)	0.59
Patient-Days with MEDD>90	2.15	0.11 (-0.30 to 0.53)	0.60	0.03 (-0.37 to 0.43)	0.87
>7 Day Opioid Fills to Opioid-Naïve Patients	0.47	0.04 (-0.02 to 0.09)	0.16	0.00 (-0.06 to 0.05)	0.93
**Days Supplied**					
All Controlled Substances	737.83	-7.55 (-23.51 to 8.40)	0.35	8.14 (-8.43 to 24.71)	0.34
Opioids	109.02	0.39 (-3.82 to 4.60)	0.86	2.62 (-1.95 to 7.18)	0.26
Benzodiazepines	76.52	-0.20 (-3.78 to 3.37)	0.91	1.21 (-2.65 to 5.08)	0.54
Gabapentinoids	238.73	-6.62 (-14.55 to 1.31)	0.10	1.34 (-6.71 to 9.38)	0.74
Stimulants	158.47	-4.13 (-11.19 to 2.92)	0.25	1.86 (-5.94 to 9.66)	0.64
OUD Medications[a]	10.20	-0.65 (-1.63 to 0.33)	0.19	-0.19 (-1.18 to 0.81)	0.72
**Equivalency Measures**					
Opioids (Morphine Mg Equivalent)	829.77	127.59 (-104.07 to 359.26)	0.28	45.36 (-181.24 to 271.96)	0.69
Benzodiazepines (Diazepam Mg Equivalent)[a]	32.11	0.18 (-1.44 to 1.81)	0.82	0.57 (-1.14 to 2.27)	0.52
Gabapentinoids (Gabapentin Mg Equivalent)[a]	10.75	-0.32 (-0.77 to 0.13)	0.17	-0.02 (-0.45 to 0.42)	0.94

* Outcomes measure prescribing during the 60 days beginning when the first e-mails were sent. Adjusted differences are least squares differences between the specified treatment group and the control group, adjusted for the outcome as measured during the 60-day baseline period and the randomization strata.

a Outcome not pre-specified in analysis plan.

PDMP: Prescription Drug Monitoring Program, BZ: Benzodiazepine, GB: Gabapentinoid, MEDD: Morphine Equivalent Daily Dose, OUD: Opioid Use Disorder, MME: Morphine Milligram Equivalents.

© 2025 Sacarny A et al. *JAMA Health Forum*

eTable 3: Effects on PDMP Engagement by Study Enrollment Group

	N	Control Mean	Legal Mandate E-mail vs. Control		Clinical Benefit E-mail vs. Control	
			Adj. Difference (95% CI)	*P* value	Adj. Difference (95% CI)	*P* value
**Has Account, %**						
By Reason for Enrollment						
No Account	4,068	2.6%	33.7 (31.0 to 36.4)	<0.001	15.3 (12.8 to 17.8)	<0.001
Inactive Account[a]	768	100.0%	0		0	
No Search[a]	1,755	100.0%	0		0	
Infrequent Search[a]	1,281	100.0%	0		0	
**Active Account, %**						
By Reason for Enrollment						
No Account	4,068	2.6%	33.8 (31.1 to 36.5)	<0.001	15.3 (12.8 to 17.8)	<0.001
Inactive Account	768	<1.4%[b]	36.3 (29.5 to 43.2)	<0.001	30.9 (24.1 to 37.7)	<0.001
No Search	1,755	>99.4%[b]	0.2 (-0.7 to 1.0)	0.70	-0.9 (-1.9 to 0.2)	0.11
Infrequent Search	1,281	>99.1%[b]	0.2 (-0.5 to 1.0)	0.53	0.5 (-0.2 to 1.1)	0.16
**Any Search, %**						
By Reason for Enrollment						
No Account	4,068	1.5%	6.7 (5.1 to 8.3)	<0.001	3.0 (1.6 to 4.5)	<0.001
Inactive Account	768	<1.4%[b]	6.8 (2.7 to 11.0)	0.001	6.7 (2.6 to 10.8)	0.001
No Search	1,755	17.1%	14.6 (9.8 to 19.5)	<0.001	8.8 (4.0 to 13.6)	<0.001
Infrequent Search	1,281	60.0%	14.4 (8.4 to 20.4)	<0.001	6.6 (0.6 to 12.7)	0.03
**Met Search Target, %**						
By Reason for Enrollment						
No Account	4,068	1.5%	6.7 (5.1 to 8.3)	<0.001	3.0 (1.6 to 4.5)	<0.001
Inactive Account	768	<1.4%[b]	6.6 (2.5 to 10.8)	0.002	6.3 (2.2 to 10.3)	0.003
No Search	1,755	17.1%	14.6 (9.8 to 19.4)	<0.001	8.7 (3.9 to 13.5)	<0.001
Infrequent Search	1,281	38.4%	13.6 (7.0 to 20.2)	<0.001	8.0 (1.3 to 14.6)	0.02
**No. of Searches**						
By Reason for Enrollment						
No Account	4,068	0.06	0.42 (0.28 to 0.56)	<0.001	0.13 (0.02 to 0.24)	0.02
Inactive Account	768	0.45	0.03 (-0.65 to 0.71)	0.93	0.29 (-0.26 to 0.84)	0.30
No Search	1,755	0.52	0.59 (0.33 to 0.85)	<0.001	0.26 (0.01 to 0.50)	0.04
Infrequent Search	1,281	4.47	1.40 (0.34 to 2.47)	0.010	0.98 (0.00 to 1.96)	0.05

* This table reports results on PDMP engagement outcomes stratified by reason for study enrollment. The outcomes measure PDMP engagement during the 60 days beginning when the first e-mails were sent. Adjusted differences are least squares differences between the specified treatment group and the control group, adjusted for the outcome as measured during the 60-day baseline period and the randomization strata. This table was not pre-specified in the analysis plan.

a Adjusted differences are zero by definition for these subgroups because all clinicians in these subgroups had already created accounts.

b Exact value suppressed for privacy.

PDMP: Prescription Drug Monitoring Program

© 2025 Sacarny A et al. *JAMA Health Forum*

eTable 4: Effects on PDMP Search and Searching for Patients with Prescribing Histories

	Control	Legal Mandate E-mail vs. Control		Clinical Benefit E-mail vs. Control	
	Mean	Adj. Difference (95% CI)	*P* value	Adj. Difference (95% CI)	*P* value
No. of Searches	0.92	0.58 (0.38 to 0.79)	<0.001	0.31 (0.12 to 0.49)	0.001
via EMR Gateway	0.65	0.28 (0.11 to 0.46)	0.001	0.12 (-0.04 to 0.27)	0.14
Issued by Main User	0.88	0.59 (0.39 to 0.79)	<0.001	0.33 (0.15 to 0.51)	<0.001
Issued by Delegate User	0.04	0.00 (-0.03 to 0.03)	0.96	-0.01 (-0.05 to 0.02)	0.38
**No. of Searches for Patients with Specified Prescribing History**[a]					
Controlled Substances					
Prescribed by Any Clinician	0.64	0.30 (0.15 to 0.46)	<0.001	0.16 (0.02 to 0.30)	0.03
Prescribed by Searcher[b]	0.40	0.18 (0.04 to 0.31)	0.009	0.10 (-0.02 to 0.22)	0.09
Opioids					
Prescribed by Any Clinician	0.46	0.23 (0.13 to 0.32)	<0.001	0.15 (0.06 to 0.25)	0.002
Prescribed by Searcher[b]	0.26	0.14 (0.06 to 0.21)	<0.001	0.11 (0.03 to 0.18)	0.005
OUD Medications[b]	0.05	0.01 (0.00 to 0.03)	0.14	0.00 (-0.02 to 0.01)	0.67
Guideline-Discordant Opioids (Any of the below)	0.31	0.17 (0.09 to 0.24)	<0.001	0.14 (0.06 to 0.21)	<0.001
Opioid-Opioid Overlap	0.12	0.05 (0.01 to 0.08)	0.01	0.04 (0.00 to 0.08)	0.05
Opioid-BZ Overlap	0.13	0.08 (0.04 to 0.13)	<0.001	0.09 (0.04 to 0.14)	<0.001
Opioid-GB Overlap	0.16	0.09 (0.05 to 0.13)	<0.001	0.06 (0.02 to 0.10)	0.004
MEDD>90 Opioids	0.01	0.00 (-0.01 to 0.01)	0.53	0.01 (0.00 to 0.02)	0.21
>7 Day Opioid Fill when Opioid-Naïve	0.05	0.04 (0.02 to 0.07)	<0.001	0.03 (0.00 to 0.05)	0.03

* Outcomes measure counts of search queries by the clinician and/or their authorized delegates during the 60 days beginning when the first e-mails were sent. Adjusted differences are least squares differences between the specified treatment group and the control group, adjusted for the outcome as measured during the 60-day baseline period and the randomization strata.

a Patients are defined as having a given prescribing history if any specified prescribing occurred in the 90 days prior to the search date. For controlled substances and for opioids, we track searches for patients with a history of receipt from any clinician and the subset of searches for patients with a history of receipt from the clinician conducting the search.

b Outcome not pre-specified in analysis plan.

EMR: electronic medical record, OUD: Opioid Use Disorder, BZ: Benzodiazepine, GB: Gabapentinoid, MEDD: Morphine Equivalent Daily Dose.

© 2025 Sacarny A et al. *JAMA Health Forum*

eTable 5: Effects on Primary Endpoints at Alternative Durations

	Control	Legal Mandate E-mail vs. Control		Clinical Benefit E-mail vs. Control	
	Mean	Adj. Difference (95% CI)	*P* value	Adj. Difference (95% CI)	*P* value
**Increased PDMP Engagement, %**[a]					
30 Days	8.8%	18.3 (16.4 to 20.3)	<0.001	8.2 (6.4 to 10.0)	<0.001
60 Days	11.8%	26.5 (24.3 to 28.7)	<0.001	14.2 (12.0 to 16.3)	<0.001
120 Days	15.9%	27.0 (24.7 to 29.3)	<0.001	15.3 (13.0 to 17.5)	<0.001
210 Days	20.5%	25.4 (23.0 to 27.8)	<0.001	14.6 (12.3 to 16.9)	<0.001
**Guideline-Discordant Prescribing**[b]					
30 Days	0.00	0.00 (-0.02 to 0.01)	0.54	0.00 (-0.01 to 0.01)	0.88
60 Days	0.00	0.00 (-0.01 to 0.01)	0.88	0.00 (-0.01 to 0.01)	0.89
120 Days	0.00	0.00 (-0.01 to 0.01)	0.76	0.00 (-0.01 to 0.01)	0.96
210 Days	0.00	0.00 (-0.01 to 0.01)	0.70	0.00 (-0.01 to 0.01)	0.93

* This table reports results on the study's two primary outcomes assessed at alternate durations (Table 2 reports the 60-day duration). The outcomes measure PDMP engagement and prescribing during the specified number of days beginning when the first e-mails were sent. Adjusted differences are least squares differences between the specified treatment group and the control group, adjusted for the outcome as measured during the 60-day baseline period and the randomization strata.
a Engagement measure differs by reason for enrollment (see Table 2).
b This outcome is the average of five standardized (mean 0, standard deviation 1) measures of guideline-discordant opioid prescribing (see Table 2).
PDMP: Prescription Drug Monitoring Program

© 2025 Sacarny A et al. *JAMA Health Forum*

eTable 6: Effects on PDMP Engagement at Alternative Durations

	Control	Legal Mandate E-mail vs. Control		Clinical Benefit E-mail vs. Control	
	Mean	Adj. Difference (95% CI)	*P* value	Adj. Difference (95% CI)	*P* value
**Has PDMP Account, %**					
30 Days	49.0%	11.6 (10.4 to 12.9)	<0.001	4.3 (3.3 to 5.4)	<0.001
60 Days	49.7%	17.4 (15.9 to 18.9)	<0.001	7.9 (6.6 to 9.2)	<0.001
120 Days	50.2%	18.0 (16.4 to 19.5)	<0.001	8.9 (7.5 to 10.3)	<0.001
210 Days	51.1%	18.2 (16.6 to 19.8)	<0.001	9.1 (7.6 to 10.6)	<0.001
**Active Account, %**					
30 Days	39.6%	14.0 (12.6 to 15.4)	<0.001	6.6 (5.3 to 7.9)	<0.001
60 Days	40.1%	21.1 (19.4 to 22.7)	<0.001	10.8 (9.3 to 12.4)	<0.001
120 Days	40.8%	21.6 (19.8 to 23.3)	<0.001	11.8 (10.1 to 13.4)	<0.001
210 Days	41.4%	21.4 (19.6 to 23.2)	<0.001	11.5 (9.8 to 13.2)	<0.001
**Any Search, %**					
30 Days	10.7%	6.0 (4.5 to 7.6)	<0.001	1.9 (0.4 to 3.4)	0.01
60 Days	14.6%	9.8 (8.1 to 11.5)	<0.001	5.3 (3.6 to 7.0)	<0.001
120 Days	20.3%	10.7 (8.9 to 12.5)	<0.001	6.7 (4.9 to 8.5)	<0.001
210 Days	24.8%	11.5 (9.5 to 13.4)	<0.001	7.8 (5.9 to 9.7)	<0.001
**Met Search Target, %**					
30 Days	8.3%	6.5 (5.0 to 8.0)	<0.001	2.3 (0.8 to 3.8)	0.002
60 Days	11.1%	9.6 (7.8 to 11.4)	<0.001	5.4 (3.6 to 7.1)	<0.001
120 Days	14.8%	11.4 (9.5 to 13.4)	<0.001	7.4 (5.5 to 9.4)	<0.001
210 Days	19.0%	12.2 (10.1 to 14.3)	<0.001	8.1 (6.1 to 10.2)	<0.001
**No. of Searches**					
30 Days	0.45	0.29 (0.18 to 0.40)	<0.001	0.08 (-0.02 to 0.18)	0.12
60 Days	0.92	0.58 (0.38 to 0.79)	<0.001	0.31 (0.12 to 0.49)	0.001
120 Days	2.11	1.26 (0.76 to 1.76)	<0.001	0.65 (0.24 to 1.06)	0.002
210 Days	4.26	2.06 (1.00 to 3.12)	<0.001	1.02 (0.13 to 1.92)	0.03

* This table reports results on PDMP engagement outcomes assessed at alternate durations (Table 2 reports the 60-day duration). The outcomes measure PDMP engagement during the specified number of days beginning when the first e-mails were sent. Adjusted differences are least squares differences between the specified treatment group and the control group, adjusted for the outcome as measured during the 60-day baseline period and the randomization strata.
PDMP: Prescription Drug Monitoring Program

© 2025 Sacarny A et al. *JAMA Health Forum*

eTable 7: Effects on PDMP Engagement, Month-by-Month

	Control	Legal Mandate E-mail vs. Control		Clinical Benefit E-mail vs. Control	
	Mean	Adj. Difference (95% CI)	*P* value	Adj. Difference (95% CI)	*P* value
**Has PDMP Account, %**					
1st Month	49.0%	11.6 (10.4 to 12.9)	<0.001	4.3 (3.3 to 5.4)	<0.001
2nd Month	49.7%	17.4 (15.9 to 18.9)	<0.001	7.9 (6.6 to 9.2)	<0.001
3rd Month	50.1%	17.8 (16.2 to 19.3)	<0.001	8.5 (7.2 to 9.9)	<0.001
4th Month	50.2%	18.0 (16.4 to 19.5)	<0.001	8.9 (7.5 to 10.3)	<0.001
5th Month	50.6%	18.2 (16.6 to 19.8)	<0.001	9.0 (7.6 to 10.5)	<0.001
6th Month	50.9%	18.2 (16.6 to 19.8)	<0.001	8.9 (7.4 to 10.4)	<0.001
7th Month	51.1%	18.2 (16.6 to 19.8)	<0.001	9.1 (7.6 to 10.6)	<0.001
**Any Search, %**					
1st Month	10.7%	6.0 (4.5 to 7.6)	<0.001	1.9 (0.4 to 3.4)	0.01
2nd Month	10.3%	7.4 (5.8 to 9.0)	<0.001	4.5 (2.9 to 6.0)	<0.001
3rd Month	11.2%	4.9 (3.3 to 6.5)	<0.001	3.8 (2.2 to 5.4)	<0.001
4th Month	12.2%	5.7 (4.0 to 7.3)	<0.001	3.1 (1.5 to 4.7)	<0.001
5th Month	12.7%	4.3 (2.7 to 6.0)	<0.001	2.8 (1.1 to 4.5)	0.001
6th Month	11.2%	4.9 (3.2 to 6.5)	<0.001	3.5 (1.9 to 5.1)	<0.001
7th Month	13.0%	4.9 (3.2 to 6.6)	<0.001	3.2 (1.5 to 4.8)	<0.001
**No. of Searches**					
1st Month	0.45	0.29 (0.18 to 0.40)	<0.001	0.08 (-0.02 to 0.18)	0.12
2nd Month	0.47	0.29 (0.16 to 0.42)	<0.001	0.23 (0.11 to 0.35)	<0.001
3rd Month	0.55	0.29 (0.13 to 0.46)	<0.001	0.15 (0.01 to 0.28)	0.03
4th Month	0.65	0.38 (0.18 to 0.59)	<0.001	0.20 (0.03 to 0.37)	0.02
5th Month	0.78	0.25 (0.01 to 0.50)	0.04	0.14 (-0.09 to 0.37)	0.23
6th Month	0.62	0.30 (0.09 to 0.51)	0.004	0.15 (-0.03 to 0.34)	0.10
7th Month	0.75	0.25 (0.02 to 0.48)	0.04	0.08 (-0.13 to 0.28)	0.46
**Guideline-Discordant Prescribing**[a]					
1st Month	0.00	0.00 (-0.01 to 0.01)	0.63	0.00 (-0.01 to 0.01)	0.79
2nd Month	0.00	0.00 (-0.01 to 0.02)	0.57	0.00 (-0.01 to 0.01)	0.85
3rd Month	0.00	0.00 (-0.02 to 0.01)	0.71	0.00 (-0.01 to 0.02)	0.61
4th Month	0.00	-0.01 (-0.02 to 0.01)	0.42	-0.01 (-0.02 to 0.01)	0.44
5th Month	0.01	-0.01 (-0.03 to 0.00)	0.16	-0.01 (-0.02 to 0.01)	0.45
6th Month	0.00	0.00 (-0.02 to 0.01)	0.69	0.00 (-0.02 to 0.02)	0.98
7th Month	0.00	0.00 (-0.01 to 0.02)	0.69	0.00 (-0.01 to 0.02)	0.74

* This table reports results on outcomes assessed only during the given month relative to the start of the interventions, with months are assumed to last 30 days. For instance, the 1st month outcomes are assessed during days 1-30 of the intervention, 2nd month outcomes are assessed during days 31-60, and so on. Adjusted differences are least squares differences between the specified treatment group and the control group, adjusted for the outcome as measured during the 60-day baseline period and the randomization strata.
a This outcome is the average of five standardized (mean 0, standard deviation 1) measures of guideline-discordant opioid prescribing (see Table 2).
PDMP: Prescription Drug Monitoring Program

© 2025 Sacarny A et al. *JAMA Health Forum*

eTable 8: Effects on Alternative Constructions of Prescribing Measures

	Control	Legal Mandate E-mail vs. Control		Clinical Benefit E-mail vs. Control	
	Mean	Adj. Difference (95% CI)	*P* value	Adj. Difference (95% CI)	*P* value
**Guideline-Discordant Overlap-Based Measures Ignoring Patients with <5 Days Overlap**					
Opioid-Opioid Overlap Days	3.38	0.12 (-0.46 to 0.71)	0.68	0.43 (-0.21 to 1.06)	0.19
Opioid-BZ Overlap Days	13.84	-0.93 (-1.84 to -0.02)	0.04	0.20 (-0.84 to 1.24)	0.70
Opioid-GB Overlap Days	32.04	-0.99 (-2.36 to 0.37)	0.15	-0.49 (-1.94 to 0.97)	0.51
**Guideline-Discordant Prescribing Measures, Measured in Distinct Patients**					
Opioid-Opioid Overlap Patients	1.00	0.01 (-0.08 to 0.10)	0.88	0.02 (-0.07 to 0.12)	0.63
Opioid-BZ Overlap Patients	1.23	-0.03 (-0.14 to 0.07)	0.55	0.05 (-0.07 to 0.16)	0.40
Opioid-GB Overlap Patients	2.08	0.01 (-0.09 to 0.11)	0.80	0.05 (-0.05 to 0.14)	0.36
Patients with 1+ day MEDD>90	0.09	0.00 (-0.02 to 0.02)	0.81	0.00 (-0.02 to 0.01)	0.63
Opioid-Naïve Patients with Opioid Fills >7 Days	0.47	0.04 (-0.02 to 0.09)	0.18	-0.01 (-0.06 to 0.05)	0.84
**Distinct Patients**					
All Controlled Substances	20.02	-0.13 (-0.56 to 0.31)	0.57	0.08 (-0.36 to 0.52)	0.72
Opioids	9.33	-0.01 (-0.35 to 0.32)	0.93	-0.07 (-0.39 to 0.26)	0.68
Benzodiazepines	2.93	0.01 (-0.13 to 0.16)	0.86	0.12 (-0.04 to 0.27)	0.15
Gabapentinoids	4.09	-0.10 (-0.23 to 0.03)	0.15	0.00 (-0.13 to 0.12)	0.95
Stimulants	2.95	-0.08 (-0.19 to 0.03)	0.13	0.06 (-0.06 to 0.18)	0.35
OUD Medications	0.28	-0.02 (-0.06 to 0.01)	0.24	-0.04 (-0.09 to 0.00)	0.08
**Fills**					
All Controlled Substances	32.13	-0.41 (-1.15 to 0.33)	0.28	0.23 (-0.53 to 0.98)	0.56
Opioids	12.58	-0.13 (-0.62 to 0.37)	0.62	-0.02 (-0.50 to 0.47)	0.94
Benzodiazepines	4.10	-0.02 (-0.22 to 0.18)	0.87	0.11 (-0.10 to 0.33)	0.31
Gabapentinoids	5.92	-0.17 (-0.37 to 0.03)	0.10	0.05 (-0.15 to 0.25)	0.64
Stimulants	5.03	-0.11 (-0.31 to 0.10)	0.31	0.11 (-0.12 to 0.34)	0.34
OUD Medications	0.48	-0.02 (-0.07 to 0.02)	0.31	-0.04 (-0.09 to 0.02)	0.21

* This table reports additional measures of prescribing quality and volume. Outcomes measure prescribing during the 60 days beginning when the first e-mails were sent. Adjusted differences are least squares differences between the specified treatment group and the control group, adjusted for the outcome as measured during the 60-day baseline period and the randomization strata. These outcomes were not pre-specified in the analysis plan.

BZ: Benzodiazepine, GB: Gabapentinoid, MEDD: Morphine Equivalent Daily Dose, OUD: Opioid Use Disorder.

© 2025 Sacarny A et al. *JAMA Health Forum*

eTable 9: Effects on Primary Endpoints by Subgroups

		Control	Legal Mandate E-mail vs. Control		Clinical Benefit E-mail vs. Control	
	N	Mean	Adj. Difference (95% CI)	*P* value	Adj. Difference (95% CI)	*P* value
**Increased PDMP Engagement, %**[a]						
Pre-Specified Subgroups						
Physician	6,574	10.1%	26.5 (24.1 to 28.9)	<0.001	14.7 (12.5 to 16.9)	<0.001
Physician Assistant	1,298	20.4%	26.7 (20.6 to 32.7)	<0.001	11.8 (6.1 to 17.5)	<0.001
No Active PDMP Account at Baseline	4,836	2.8%	34.1 (31.7 to 36.6)	<0.001	17.8 (15.6 to 19.9)	<0.001
Active PDMP Account at Baseline	3,036	26.1%	14.1 (10.0 to 18.1)	<0.001	8.4 (4.4 to 12.4)	<0.001
By Reason for Enrollment (Outcome)[b]						
No Account (Has Account, %)	4,068	2.6%	33.7 (31.0 to 36.4)	<0.001	15.3 (12.8 to 17.8)	<0.001
Inactive Account (Active Account, %)	768	<1.4%[c]	36.3 (29.5 to 43.2)	<0.001	30.9 (24.1 to 37.7)	<0.001
No Search (Any Search, %)	1,755	17.1%	14.6 (9.8 to 19.5)	<0.001	8.8 (4.0 to 13.6)	<0.001
Infrequent Search (Met Search Target, %)	1,281	38.4%	13.6 (7.0 to 20.2)	<0.001	8.0 (1.3 to 14.6)	0.02
**Guideline-Discordant Prescribing**[d]						
Pre-Specified Subgroups						
Physician	6,574	0.00	0.00 (-0.01 to 0.01)	0.98	0.00 (-0.01 to 0.02)	0.75
Physician Assistant	1,298	-0.02	0.00 (-0.02 to 0.02)	0.83	0.00 (-0.03 to 0.02)	0.71
No Active PDMP Account at Baseline	4,836	-0.09	0.00 (-0.01 to 0.01)	0.58	0.00 (-0.01 to 0.02)	0.67
Active PDMP Account at Baseline	3,036	0.15	0.00 (-0.02 to 0.02)	0.77	0.00 (-0.02 to 0.02)	0.88
By Reason for Enrollment[b]						
No Account	4,068	-0.09	0.00 (-0.01 to 0.01)	0.75	0.00 (-0.01 to 0.01)	0.99
Inactive Account	768	-0.09	0.01 (-0.02 to 0.03)	0.69	0.02 (-0.01 to 0.06)	0.24
No Search	1,755	-0.03	0.00 (-0.03 to 0.02)	0.81	0.00 (-0.02 to 0.02)	0.89
Infrequent Search	1,281	0.39	-0.01 (-0.05 to 0.03)	0.62	-0.01 (-0.06 to 0.03)	0.53

* This table reports results on the study's two primary outcomes by subgroups. The outcomes measure PDMP engagement and prescribing during the 60 days beginning when the first e-mails were sent. Adjusted differences are least squares differences between the specified treatment group and the control group, adjusted for the outcome as measured during the 60-day baseline period and the randomization strata.
a Engagement measure differs by reason for enrollment (see Table 2). When analyzing this outcome by reason for enrollment, the text indicates the engagement measure in parentheses.
b Subgroups not pre-specified in analysis plan.
c Exact value suppressed for privacy.
d This outcome is the average of five standardized (mean 0, standard deviation 1) measures of guideline-discordant opioid prescribing (see Table 2).
PDMP: Prescription Drug Monitoring Program

© 2025 Sacarny A et al. *JAMA Health Forum*
