# Supplementary material for: Prescription Drug Monitoring Program Reminder Emails, Program Use, and Prescribing: A Randomized Clinical Trial
Source: JAMA Health Forum. 2025 Dec 19;6(12):e255623. doi: 10.1001/jamahealthforum.2025.5623 (PMC12717620; doi:10.1001/jamahealthforum.2025.5623)
Supplement: Supplement 3. — Data Sharing Statement [file jamahealthforum-e255623-s003.pdf]

# Data Sharing Statement

Sacarny. Prescription Drug Monitoring Program Reminder Emails, Program Use, and Prescribing. *JAMA Health Forum*. Published December 19, 2025.
doi:10.1001/jamahealthforum.2025.5623

**Data**
**Additional Information:** ClinicalTrials.gov NCT06443385
**Data available:** No

**Additional Information**
**Explanation for why data not available:** This study uses data provided by the Minnesota Prescription Monitoring Program. Due to statutory restrictions on the release of this data, we cannot make individual patient- or clinician-level records publicly available.
